# Stable Isotope Probing Identifies Bacterioplankton Lineages Capable of Utilizing Dissolved Organic Matter Across a Range of Bioavailability

**DOI:** 10.3389/fmicb.2020.580397

**Published:** 2020-10-07

**Authors:** Shuting Liu, Nicholas Baetge, Jacqueline Comstock, Keri Opalk, Rachel Parsons, Elisa Halewood, Chance J. English, Stephen Giovannoni, Luis M. Bolaños, Craig E. Nelson, Kevin Vergin, Craig A. Carlson

**Affiliations:** ^1^Department of Ecology, Evolution, and Marine Biology, Marine Science Institute, University of California, Santa Barbara, Santa Barbara, CA, United States; ^2^Bermuda Institute of Ocean Sciences, Saint George, Bermuda; ^3^Department of Microbiology, Oregon State University, Corvallis, OR, United States; ^4^Daniel K. Inouye Center for Microbial Oceanography: Research and Education, Department of Oceanography and Hawai‘i Sea Grant, School of Ocean and Earth Science and Technology, University of Hawai‘i at Mānoa, Honolulu, HI, United States; ^5^Microbial DNA Analytics, Phoenix, OR, United States

**Keywords:** stable isotope probing, labile, recalcitrant, DOM, bioavailability, copiotrophs, oligotrophs, Sargasso Sea

## Abstract

Bacterioplankton consume about half of the dissolved organic matter (DOM) produced by phytoplankton. DOM released from phytoplankton consists of a myriad of compounds that span a range of biological reactivity from labile to recalcitrant. Linking specific bacterioplankton lineages to the incorporation of DOM compounds into biomass is important to understand microbial niche partitioning. We conducted a series of DNA-stable isotope probing (SIP) experiments using ^13^C-labeled substrates of varying lability including amino acids, cyanobacteria lysate, and DOM from diatom and cyanobacteria isolates concentrated on solid phase extraction PPL columns (SPE-DOM). Amendments of substrates into Sargasso Sea bacterioplankton communities were conducted to explore microbial response and DNA-SIP was used to determine which lineages of Bacteria and Archaea were responsible for uptake and incorporation. Greater increases in bacterioplankton abundance and DOC removal were observed in incubations amended with cyanobacteria-derived lysate and amino acids compared to the SPE-DOM, suggesting that the latter retained proportionally more recalcitrant DOM compounds. DOM across a range of bioavailability was utilized by diverse prokaryotic taxa with copiotrophs becoming the most abundant ^13^C-incorporating taxa in the amino acid treatment and oligotrophs becoming the most abundant ^13^C-incorporating taxa in SPE-DOM treatments. The lineages that responded to SPE-DOM amendments were also prevalent in the mesopelagic of the Sargasso Sea, suggesting that PPL extraction of phytoplankton-derived DOM isolates compounds of ecological relevance to oligotrophic heterotrophic bacterioplankton. Our study indicates that DOM quality is an important factor controlling the diversity of the microbial community response, providing insights into the roles of different bacterioplankton in resource exploitation and efficiency of marine carbon cycling.

## Introduction

Bacterioplankton incorporate dissolved organic matter (DOM) as their nutrient and energy resources, transform DOM via biotic processes, and mediate the transfer of energy and nutrients between trophic levels (Azam et al., 1983; Jiao et al., 2010; Bertrand et al., 2015). Extracellular release from phytoplankton is one of the major DOM production processes and heterotrophic bacterioplankton consume roughly half of the DOM produced by daily primary production (Williams, 1981). DOM released from phytoplankton consists of a myriad of compounds that span a range of turnover times and biological reactivity from labile to recalcitrant (Carlson, 2002; Hansell, 2013; Carlson and Hansell, 2015). A number of studies have demonstrated that the rate, efficiency, and composition of bacterioplankton growth is sensitive to the source of phytoplankton DOM, including effects of phytoplankton community structure (Pinhassi and Berman, 2003; Teeling et al., 2012), phytoplankton bloom dynamics (Wear et al., 2015), ocean acidification (James et al., 2019) and nutrient regime (Goldberg et al., 2017). Identifying how components of the DOM pool released by phytoplankton are incorporated by different bacterioplankton taxa is central to understanding both the dynamics of DOM globally and the factors maintaining the tremendous diversity of heterotrophic bacterioplankton in the world’s oceans.

Solid phase extraction (SPE) via hydrophobic resin, such as XAD, C-18, and PPL (Priority Pollutant), has often been used to isolate a fraction of DOM from seawater (Mopper et al., 2007; Dittmar et al., 2008). The hydrophobicity of most SPE columns results in low retention of certain compounds, such as highly polar organic acids. In fact, depending on the SPE resin and sample matrix (i.e., seawater, freshwater, cultures, etc.), only ∼10−60% of bulk DOM is retained on the columns (Meyers-Schulte and Hedges, 1986; Dittmar et al., 2008; Swenson et al., 2014; Johnson et al., 2017). Humic substances retained on and extracted from SPE columns are rich in carboxyl groups and aliphatic carbon and contain a class of persistent DOM (Druffel et al., 1992; Hedges et al., 1992; Lechtenfeld et al., 2014). Microbial remineralization experiments indicate that SPE-DOM from natural systems is dominated by recalcitrant compounds that are resistant to rapid microbial degradation (Shen and Benner, 2018, 2019). This suggests that pretreatment of bulk DOM by concentrating on SPE columns can be used to alter DOM quality. Empirically, DOM quality is a function of complex interactions between the biochemical characteristics of DOM molecules and the metabolic capabilities of diverse members of microbial communities (Nelson and Wear, 2014). Despite this complexity, it appears that the compounds enriched by SPE can broadly be classified as low quality, recalcitrant material.

Dissolved organic matter composition has been shown to shape bacterioplankton community structure, potentially due to variation in trophic strategies among taxa of heterotrophic plankton (Cottrell and Kirchman, 2000; Yooseph et al., 2010; Nelson and Carlson, 2012; Nelson et al., 2013; Landa et al., 2016). Bacterioplankton are typically categorized as oligotrophs or copiotrophs, adapted to low and high nutrient concentrations, respectively (Lauro et al., 2009; Yooseph et al., 2010). While oligotrophs are typically small, slow-growing cells with small genomes, copiotrophs are typically larger cells that use a “feast or famine” strategy to adapt to rapidly changing environments (Eilers et al., 2000; Giovannoni et al., 2005). For example, the abundant and ubiquitously distributed bacterioplankton SAR11 (*Pelagibacterales*) with streamlined genomes are representative oligotrophs while the larger *Roseobacter* and *Alteromonadaceae* are examples of copiotrophs (Polz et al., 2006; Buchan et al., 2014; Pedler et al., 2014; Giovannoni, 2017). Based on the niche width, bacterioplankton can also be characterized as generalists or specialists. While environmental heterogeneity favors selection of generalists that can utilize a diverse group of substrates, specialists evolve in homogeneous environments and have narrow niche width (Kassen, 2002; Mou et al., 2008). For instance, members of the *Bacteroidetes* and *Flavobacteria* clades are specialized in utilizing high molecular weight (HMW) polymers, whereas members of the *Roseobacter* clade exhibit diverse metabolic capabilities of nutrient acquisition (Teira et al., 2009; Zheng et al., 2019b). Understanding the interaction between DOM and bacterioplankton with varying trophic strategies and niche breadth is key to gaining insight into niche partitioning and resource competition between bacterioplankton populations.

Numerous seawater incubation and field studies have simultaneously measured the change in bacterioplankton biomass or activity in the context of organic matter utilization or have used molecular approaches like fluorescence *in situ* hybridization (FISH), catalyzed reporter deposition (CARD)-FISH, and amplicon sequencing to resolve changes of specific bacterioplankton lineages when DOM resources were introduced (Eilers et al., 2000; Harvey et al., 2006; Liu et al., 2020). While these studies provide potential linkages between DOM uptake and specific microbial response, the relationship between DOM and bacterioplankton phylogeny is only correlative, lacking direct evidence of uptake of specific DOM components into bacterioplankton biomass. Tracking organic matter labeled with stable isotopes into bacterioplankton biomass via stable isotope probing (SIP) is a promising procedure to better understand DOM metabolism and directly track utilization of targeted DOM components into the biomass of responding microbial populations (Neufeld et al., 2008; Mayali et al., 2014; Seyler et al., 2019). The principle of SIP is to amend an experimental incubation with a substrate labeled with heavy stable isotope (i.e., ^13^C or ^15^N) and track the labeled compounds into cellular biomass components, like phospholipid-derived fatty acid, DNA or RNA. Using an extended ultracentrifugation, the cellular components labeled by the heavy isotope are separated along a CsCl density gradient, isolated, and sequenced, thus linking phylogeny of organisms to the uptake of specific substrates or to specific metabolic function (Radajewski et al., 2003; Neufeld et al., 2007c). In marine environments, SIP has been used extensively in previous studies to identify bacterioplankton taxa incorporating simple model compounds or labile DOM sources such as peptides, proteins, amino acids, urea, and methane (Redmond et al., 2010; Connelly et al., 2014; Mayali et al., 2014; Orsi et al., 2016; Liu et al., 2017). However, one of the challenges of applying SIP to study natural microbial assemblages is that these model compounds or freshly derived complex organic matter can be highly labile and often disproportionately enrich for copiotrophs that may not be representative of bacterioplankton capable of utilizing more recalcitrant compounds that are present in the environment. To date, only a few studies have used the DNA-SIP approach to compare and contrast the responding bacterioplankton community capable of incorporating DOM of varying quality (Nelson and Carlson, 2012; Bryson et al., 2017); in particular using this approach to identify bacterioplankton capable of incorporating recalcitrant/transformed DOM has not been well explored.

In the northwestern Sargasso Sea, annual winter convective mixing redistributes semi-labile and semi-refractory DOM accumulated in the summer-autumn stratified period from surface to the upper mesopelagic water (Carlson et al., 1994; Hansell and Carlson, 2001), leading to a responding bacterioplankton community of oligotrophs, including members of the *Alphaproteobacteria* SAR11 (now order *Pelagibacterales*), the *Chloroflexi* SAR202 (now class *Monstramaria*), and the *Alphaproteobacteria* OCS116 clades that may be capable of utilizing recalcitrant DOM (Carlson et al., 2009; Treusch et al., 2009; Landry et al., 2017; Saw et al., 2020). In this study, we altered the quality of ^13^C-labeled DOM substrates to explore if the alteration might select for responding microbes representative of those observed in the mesopelagic of the Sargasso Sea during or following the downward export of surface DOM via convective mixing. We conducted a series of DNA-SIP experiments focused on microbial assemblages collected from the euphotic and mesopelagic zones of the northwestern Sargasso Sea to explore which lineages of bacterioplankton were capable of responding to and incorporating a suite of organic compounds of varying complexity and lability. The ^13^C labeled DOM ranged from model organic compounds (i.e., amino acids) to phytoplankton derived ^13^C labeled DOM lysates and exudates. PPL columns were also used to further alter the quality of phytoplankton derived DOM. Using various DOM substrates, we aimed to compare bacterioplankton responses to (1) labile model compounds and complex DOM (amino acids vs. phytoplankton-derived DOM), (2) PPL-altered vs. unaltered DOM (*Synechococcus* lysate vs. lysate_*PPL*_, and exudate_*PPL*_), (3) phytoplankton DOM of different origins (*Synechococcus* vs. *Thalassiosira weissflogii* DOM), and to compare the responses of (4) surface and mesopelagic bacterioplankton to DOM additions (more details below).

## Materials and Methods

### Phytoplankton Culturing, DOM Substrate Extraction, and Characterization

A cyanobacteria and diatom were chosen as representative phytoplankton species to produce complex phytoplankton-derived ^13^C labeled DOM. *Synechococcus* (Syn) is an important phytoplankton group representing ∼9−23% of phytoplankton biomass in the Sargasso Sea (Olson et al., 1990; Durand et al., 2001). Diatoms contribute to 25−75% of global ocean primary production and *Thalassiosira weissflogii* (TW) is an important coastal marine diatom species (Nelson et al., 1995). Diatoms are rare but occasionally bloom in the Sargasso Sea during springtime (Siegel, 1990; Krause et al., 2009). Although accounting for only <5% of total chlorophyll (Goericke, 1998), diatoms can contribute up to ∼25% of new production and 41−100% of particulate organic carbon (POC) export flux from the upper 200 m in response to late-winter storms in the Sargasso Sea (Krause et al., 2009).

TW (strain CCMP1336, from Bigelow Laboratory for Ocean Sciences) and Syn (strain CC9902, isolated from Santa Barbara Channel and inferred from NCBI Blast) were cultured in f/2 and L1 media, respectively (Guillard and Ryther, 1962; Guillard, 1975; Guillard and Hargraves, 1993). Trace metal concentrations in EDTA solution were reduced to 10% of recommended values in order to minimize organic matter contribution associated with the chelator solution. Media was amended with ^13^C-sodium bicarbonate (final concentration of 2.38 mmol L^–1^) in artificial seawater (ASW, 420 mmol L^–1^ NaCl, 28.8 mmol L^–1^ Na_2_SO_4_, 9.39 mmol L^–1^ KCl, 0.84 mmol L^–1^ KBr, 0.0485 mmol L^–1^ H_3_BO_3_, 0.0715 mmol L^–1^ NaF, 54.6 mmol L^–1^ MgCl_2_.6H_2_O, 10.5 mmol L^–1^ CaCl_2_.2H_2_O, 0.0638 mmol L^–1^ SrCl_2_.6H_2_O) and cultures were grown at 14°C with a 14:10 light/dark cycle. After TW cell density reached approximately 10^8^ cells/L and Syn reached approximately 10^10^ cells/L, cultures were harvested and multiple 250 ml aliquots were centrifuged at 10,000 rpm (6,684 × *g*) for 15 min (Nelson and Carlson, 2012). The supernatant was filtered through combusted GF/F (Whatman) then through a pre-rinsed 142 mm 0.2 μm pore-size polyethersulfone filter (Supor) and the resulting filtrate was characterized as culture “exudate.” Next the cell pellets were washed three times by resuspending in 60 mL ASW (no added nutrients) and re-pelleting at 10,000 rpm for 15 min to remove excess media. The pellets were then placed in 5 mL Nanopure water and stored frozen at −20°C. This slurry was thawed and frozen three times and then vortexed for at least 5 min with 0.5 mL pre-combusted garnet beads (MoBio Laboratories) to ensure rupturing of cells and release of cytosol. The slurry was aliquoted into multiple 2 mL microcentrifuge tubes and spun at 13,000 rpm (12,470 × *g*) for 20 min. Supernatant was filtered through combusted GF/F and then syringe filtered through a pre-rinsed 0.2 μm polycarbonate filter. The final filtrate is referred to as phytoplankton “lysate.”

To further alter the quality of lysate and exudate DOM and also remove any inorganic nutrients carried from the culture media, a portion of the phytoplankton-derived DOM lysate and exudate was extracted using Bond Elut PPL cartridges (Agilent 1 g/5 mL) and solid phase extraction method following the protocol by Dittmar et al. (2008). Extracts from PPL cartridges were eluted with 8 mL methanol, dried under N_2_ gas and resuspended in 8 mL Nanopure water. We refer to the final extracts as “lysate_*PPL*_” and “exudate_*PPL*_.” PPL cartridges were found to be more efficient than C-18 in extracting dissolved organic carbon (DOC) from seawater, retaining approximately 43−65% of DOC in oceanic seawater (Dittmar et al., 2008). However, we observed an 8−15% recovery of DOC after PPL extraction of our phytoplankton lysates and exudates.

To compare the effects of PPL extraction on the chemical quality of DOM substrates, 0.5 mL ^13^C-Syn lysate and ^13^C-Syn lysate_*PPL*_ extract stock were lyophilized and dissolved in dimethyl sulfoxide-D6 (Cambridge Isotope Laboratories, 99.9% D) for 2D [^13^C, ^1^H] heteronuclear single quantum coherence (HSQC) nuclear magnetic resonance (NMR) analysis. Eight to twelve scans were performed on samples to identify major functional groups containing ^13^C and correlation with ^13^C-attached H. Analysis were conducted on a Varian Unity Inova 500 MHz at the University of California Santa Barbara (UCSB) NMR facility (Department of Chemistry and Biochemistry) following established protocol (Davis, 1991; Schmieder et al., 1991; Davis et al., 1992).

Urea concentrations in Syn lysate, Syn lysate_*PPL*_, and Syn exudate_*PPL*_ substrates were measured using the room-temperature diacetylmonoxime method on a spectrophotometer (Goeyens et al., 1998).

### Incubation Setup and Sampling

Seawater for DOM remineralization experiments was collected from the surface (10 m) and/or mesopelagic (200 m) zones in the vicinity of Hydrostation S (HS) (32°10′N, 64°30′W) or the Bermuda Atlantic Time-series Study (BATS) spatial station (SS#1) (31°46′N, 64°43′W) in the northwestern Sargasso Sea during thermally stratified periods of July (2016, 2017) and November (2017). Seawater was sampled via Niskin bottles on a conductivity, temperature, and depth profiling rosette onboard the R/V *Atlantic Explorer*. Incubation media was generated by mixing unfiltered whole seawater with 0.2 μm seawater filtrate generated by gravity filtration through a polycarbonate or mixed cellulose ester (pre-rinsed with 2 liters of milli-Q water and seawater) at a ratio of 30:70. Three sets of DOM substrates were amended to the surface and/or mesopelagic medium for incubation during three cruises (Table 1): ^13^C-TW lysate_*PPL*_ extract; ^13^C-amino acid mixture (L-alanine-1-^13^C with hydrophobic side chain, L-serine-1-^13^C with polar side chain and L-methionine-carboxy-^13^C with sulfur-containing side chain at equal concentrations; Sigma-Aldrich; 99% ^13^C); ^13^C-Syn lysate, ^13^C-Syn lysate_*PPL*_ extract, ^13^C-Syn exudate_*PPL*_ extract. Free amino acid monomers were chosen as representative labile model compounds to contrast complex phytoplankton-derived DOM; three Syn DOM substrates were designed to directly compare bacterioplankton response between PPL-altered and non-altered DOM; TW DOM was introduced as a substrate from a different phytoplankton origin to compare with Syn DOM. The majority of the experimental treatments were conducted with mesopelagic seawater in order to simulate the response of bacterioplankton to DOM export during the annual convective mixing event in the Sargasso Sea (Carlson et al., 1994; Hansell and Carlson, 2001) and also to use seawater that was not limited by the availability of inorganic macro nutrients (Steinberg et al., 2001), with the exception that TW DOM incubation was conducted in both surface and mesopelagic seawater for a comparison between different ambient conditions. The isotope values of Syn substrates were measured at the University of California Davis Stable Isotope Facility using a TOC analyzer (Xylem Analytics, College Station, TX, United States) interfaced to an isotope ratio mass spectrometer (Sercon Ltd., Cheshire, United Kingdom). Syn lysate, Syn lysate_*PPL*_, and Syn exudate_*PPL*_ were 100 atom%, 64 atom%, and 44 atom% ^13^C-labeled, respectively, and TW lysate_*PPL*_ with similar extraction efficiency on PPL columns was assumed to have a similar range (44−64%) of ^13^C labeling.

**TABLE 1:** Amended ^13^C-DOM substrates and experimental incubation details.

Experiment Time	Station	Latitude, Longitude	Treatments	Water depth (m)	*In situ* temperature (°C)	Amended DOC (μmol C L^–1^)	Amended TDAA C (μmol C L^–1^)	TDAA C yield in substrate (%)	DNA harvesting time (d)
July 2016	HS	32°10′N, 64°30′W	S^*a*^ control	10	27.0	−	−	−	2.4
			S TW^b^ lysate_*PPL*_^c^	10	27.0	6.2	3.98	64	
			M^d^ control	200	20.0	−	−	−	
			M TW lysate_*PPL*_	200	20.0	*6.0*^f^	3.85	64^f^	
July 2017	HS	32°10′N, 64°30′W	M control	200	19.1	−	−	−	4.3
			M Amino acid	200	19.1	3.8	−	−	
November 2017	SS#1	31°46′N, 64°43′W	M control	200	19.7	−	−	−	2.7
			M Syn^e^ lysate	200	19.7	4.8	0.69	15	
			M Syn lysate_*PPL*_	200	19.7	2.0	1.11	57	
			M Syn exudate_*PPL*_	200	19.7	4.1	1.11	27	

*^*a*^S, surface. ^*b*^TW, *Thalassiosira Weissfloggi*. ^*c*^PPL, PPL cartridge extracts. ^*d*^M, mesopelagic. ^*e*^Syn, *Synechococcus*. ^*f*^The measured DOC value for the mesopelagic treatment was measured at 1.7 μM C instead of the expected 6.2 μM resolved to the surface treatment and appears to have been underestimated due to preservation and storage problems. The DOC value in italic represents the calculated concentration based on measured TDAA-C of the amended substrate and assuming same TDAA C yield observed for the surface amendment of the 2016 experiment. See “Results” section for details.*

Dissolved organic matter substrates were amended at final concentrations of 2−6 μmol C L^–1^ into duplicate 5 L polycarbonate carboys filled with seawater medium (30:70 mixture as described above) and incubated in the dark at *in situ* temperatures in upright incubators (Thermo Fisher Scientific Isotemp incubators) for 2−4 days until bacterioplankton reached late exponential to stationary growth phase. Unamended control treatments with only seawater medium were also performed. Bacterioplankton abundance (BA), DOC and total dissolved amino acids (TDAA) replicates were sampled at regular time intervals from duplicate carboys. DNA was sampled at the initial time point (T0) and final harvesting time point (TF) of the incubation. BA (10 mL) samples were fixed with 0.2 μm filtered formaldehyde (1% final concentration), stored at 4°C and processed within 48 h or stored at −80°C until slide preparation. DOC samples (duplicate 30 mL draws) were filtered through double-stacked combusted GF-75 filters (Advantec, 0.3 μm pore-size, dia. 25 mm) into combusted 40 mL EPA glass vials and acidified to pH < 3 with 4N HCl. TDAA samples (30 mL) were filtered through the same double-stacked GF-75 filters into 60 mL acid-washed high density polyethylene (HDPE) bottles and stored at −20°C. 500 mL from each replicate treatment was combined (total 1 L) and filtered through an Omnipore polytetrafluoroethylene (PTFE) filter (0.2 μm pore-size, dia. 47 mm) for T0 DNA collection. Approximately 1 to 3.5 L of water from each incubation carboy was filtered on Omnipore filters and replicate treatments were pooled (total 2−7 L filtered) to obtain enough DNA at the final harvesting time point for DNA-SIP fractionation (described below). DNA filters were stored at −80°C in sucrose lysis buffer [750 mmol L^–1^ sucrose, 20 mmol L^–1^ ethylenediaminetetraacetic acid (EDTA), 400 mmol L^–1^ NaCl, 50 mmol L^–1^ Tris−HCl, pH 8.0] (Giovannoni et al., 1990).

### BA Counting, Biovolume, and Bacterial Carbon

Prokaryotic cells were stained with 5 μg mL^–1^ 4′,6-diamidino-2-phenylindole dihydrochloride (DAPI, Sigma-Aldrich) and enumerated with an Olympus AX70 epifluorescence microscope under ultraviolet excitation at 1000 × magnification (Porter and Feig, 1980). As this counting cannot differentiate between Bacteria and Archaea, bacterioplankton henceforth refers to both Bacteria and Archaea. Cell biovolume for each sample was based on 10 images captured with a digital camera (Retiga Exi-QImaging, Surrey, BC, Canada) and analyzed with ImageJ software. Cell length (L, major axis) and width (W, minor axis) were obtained from image analysis and biovolume (V) was calculated as:

(1) $$\text{V}=\pi \,\left(\text{L}-\text{W}\right)\,{\text{W}}^{2}/4+\pi \,W^{3}/6$$

when L/W > 1.5 it is assumed that the cell shape is a cylinder with hemispherical ends or:

(2) $$\text{V}=\pi \,W^{3}/6$$

when L/W < 1.5 it is assumed that the cell shape is spherical (Baldwin and Bankston, 1988; Sieracki et al., 1989). Bacterial carbon (BC) was calculated as:

(3) BC=BA×cell⁢biovolume×CCF

where CCF is the carbon conversion factor of 148 fg C μm^–3^ (Gundersen et al., 2002).

### DOC Analysis

Dissolved organic carbon was analyzed using the high temperature catalytic oxidation (HTCO) method on a modified TOC-V or TOC-L analyzer (Shimadzu) in the shore based laboratory at UCSB (Carlson et al., 2010). Combustion tubes were extensively conditioned with low-carbon Nanopure water and deep seawater to minimize instrument blanks and achieve a stable baseline. The CO_2_ in the carrier gas was analyzed with a non-dispersive infrared detector and the resulting peak area was integrated with Shimadzu chromatographic software. Glucose standards (25−100 μmol C L^–1^) and reference seawaters were used daily for calibration. The reference seawaters were previously calibrated with DOC Consensus Reference Material (CRM) provided by D. Hansell (University of Miami) (Hansell, 2005). The precision for DOC analysis is ∼1 μmol L^–1^ or a CV of ∼2%.

### TDAA Analysis

Replicate TDAA samples were hydrolyzed in 6N HCl under nitrogen at 110°C for 20 h and then neutralized using nitrogen evaporation (Henrichs, 1991). Nanopure blanks followed the same extraction protocol. TDAA were derivatized with *o*-phthaldialdehyde and measured by high performance liquid chromatography (HPLC, Dionex ICS5000+) equipped with a fluorescence detector (Ex = 330 nm, Em = 418 nm) following the established gradient program (Kaiser and Benner, 2009; Liu et al., 2013, 2020). The molecular formula for each AA that was resolved by the HPLC analyses was used to calculate the individual AA concentration in carbon units. The TDAA carbon (TDAA C) represents the sum of all of the individual AA concentrations in carbon units.

### DNA Extraction and CsCl Gradient Fractionation

DNA was extracted using phenol and chloroform following the protocol of Giovannoni et al. (1996). For the final harvesting time point, 20 μL DNA (11−975 ng) was used as an unfractionated sample for whole microbial community structure analysis and the remaining 80 μL (45−3,900 ng) was used for fractionation along a CsCl density gradient following the protocol described in Neufeld et al. (2007a) and modified by Nelson and Carlson (2012). In brief, DNA was mixed with gradient buffer (autoclaved 100 mmol L^–1^ Tris−HCl, 100 mmol L^–1^ KCl and 1 mmol L^–1^ EDTA) and CsCl solution (autoclaved 1.85 g mL^–1^ in gradient buffer) to a final density of approximately 1.73 g mL^–1^ and transferred into 3.3 mL OptiSeal tubes (Beckman Coulter). This mixture was centrifuged at 177,000 × *g* in a near-vertical rotor (Beckman TLN-100) under vacuum and at 20°C in a Beckman Optima Max-XP ultracentrifuge for 40 h. Gradients were fractioned into ten 325 μL fractions by syringe pumping sterile Nanopure water at the top and collecting drops from the bottom of the tube. Each fraction was mixed with 2 volumes of 30% polyethylene glycol (PEG6000) and 1 μL glycogen (20 mg mL^–1^) for DNA precipitation. After 2 h, samples were centrifuged at 16,000 × *g* for 30 min and supernatant was aspirated. Then samples were washed with 70% ethanol, centrifuged again and pellets were dried at 37°C and resuspended in DNase-free sterile deionized water.

### DNA Library Preparation, Illumina Sequencing, and Bioinformatics

Genomic DNA was amplified with 16S rRNA gene V4 primer 515F (5′-GTGYCAGCMGCCGCGGTAA-3′) and 806R (5′-GGACTACNVGGGTWTCTAAT-3′) with custom adapters (Wear et al., 2018). Amplicons were purified and normalized to ∼ 6−14 ng with SequalPrep plates (Invitrogen) and pooled at equal volumes. The pooled library was concentrated by the Amicon Ultra 0.5 ml 30 kDa filters (Millipore), gel-extracted to remove non-target bands (Qiagen Qiaquick) and sequenced at the University of California, Davis DNA Technologies Core on an Illumina MiSeq using PE250.

Sequence data were trimmed, dereplicated, amplicon sequence variants (ASV) determined, chimera checked, and taxonomic assigned using the DADA2 R package version 1.2 and SILVA v132 database (Callahan et al., 2016). For finer phylogenetic taxonomy assignment of the SAR11 and SAR202 clade, sequences were further run through the PhyloAssigner program (Vergin et al., 2013a). Sequences are deposited in the National Center for Biotechnology Information (NCBI) Sequence Read Archive (SRA) under project number PRJNA577154.

### Identification and Validation of Bacterioplankton Incorporating ^13^C

**FIGURE 1:**
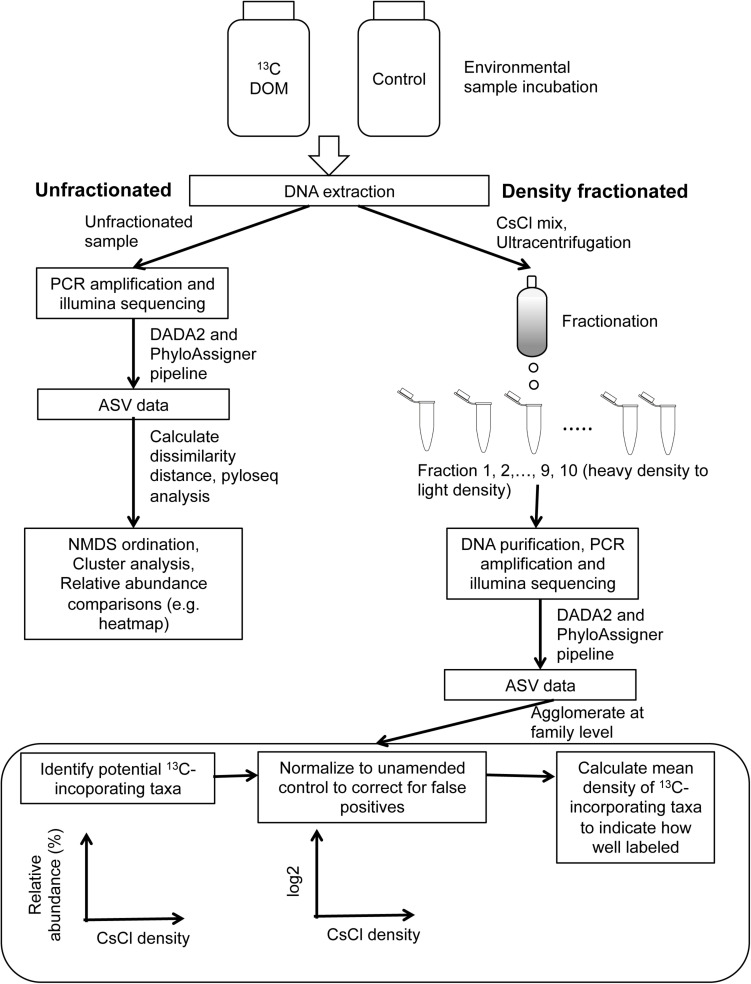
Flow chart of SIP sample processing and data analysis steps.

A complete conceptual diagram for SIP sample processing and data analysis is presented in Figure 1. To identify taxa that incorporate ^13^C-DOM, 16S rRNA gene relative abundance across a CsCl density gradient was plotted for major family taxa (>0.1% in at least one fraction) (Supplementary Figure 1). Because fraction 1 (heaviest fraction) and fraction 10 (lightest fraction) contain mostly CsCl or sterile water and very few DNA sequences, they were excluded for further analysis. Theoretically, a population of cells that is well-labeled with ^13^C will have higher relative abundance in the heavy (high density) fractions compared to lighter fractions. Taxa of unlabeled populations will be most pronounced in the lighter CsCl fractions. A population of cells that is partially labeled will result in a higher relative abundance observed in both the high and low density fractions compared to medium density fractions (Supplementary Figure 1A; Nelson and Carlson, 2012). However, due to variable GC contents among various taxa and potential PCR amplification of trace contaminants, it is possible that unlabeled DNA will also smear across density fractions (Buckley et al., 2007; Neufeld et al., 2007b). Thus, to account for the unlabeled contribution of any given taxon, DNA samples of the unamended controls were also prepared in a CsCl gradient and fractionated in parallel to the amended samples. The relative abundance of one taxon in the amended treatment was divided by that in the unamended control treatment across all fractions, log2 transformed, and plotted against CsCl densities (Supplementary Figure 2). Three criteria were used to identify whether one family level taxon was incorporating ^13^C: (1) relative abundance of a taxon was >0.0001% (2/minimal reads) in at least three fractions; (2) relative abundance was >0.1% in unamended control unfractionated community or amended unfractionated community to avoid very rare populations and associated PCR bias; (3) relative abundance of a taxon showed “well-labeled” or “partially-labeled” pattern with CsCl density and log2 transformed relative abundance over unamended control did not show ambiguous, uniform or “unlabeled” pattern across various CsCl densities (Supplementary Figures 1, 2). As 20% taxa labeling is needed to confidently identify ^13^C incorporation, a threshold of 0.53 [i.e., ±log_2_ (1 + 20%)] in the log_2_(^13^C/unamended) range between lowest and highest values was used to identify significant change across density fractions (Uhlik et al., 2009).

**FIGURE 2:**
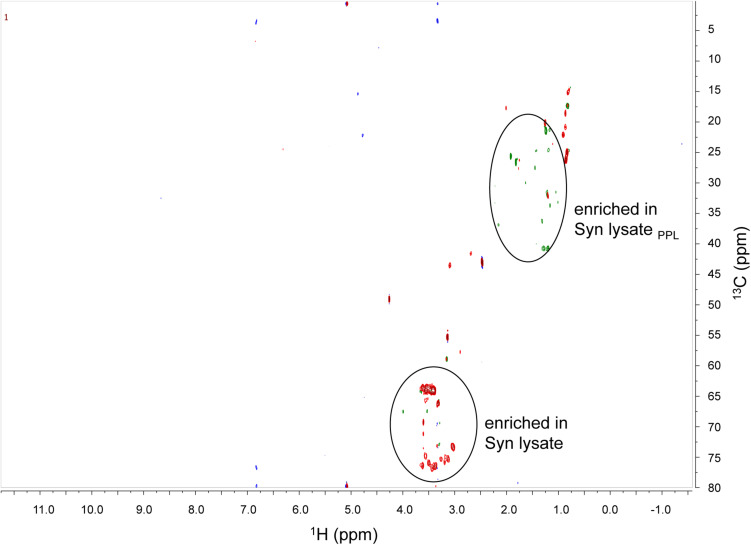
NMR 2D ^13^C-^1^H HSQC plots (^1^H on x axis and ^13^C on y axis) of superimposed ^13^C Syn lysate (red positive contours) and ^13^C Syn lysate_*PPL*_ (green positive contours) samples in deuterated DMSO. Noise negative contours are in blue. Circles are functional groups enriched in Syn lysate or Syn lysate_*PPL*_ samples.

To verify that our method of CsCl density fractionation and identification of ^13^C incorporators can differentiate labeled from unlabeled bacterioplankton without confounding natural GC content variation, we conducted incubations using ^12^C TW lysate_*PPL*_ extracts along with ^13^C TW lysate_*PPL*_ extracts incubations in the July 2016 experiments. ^12^C TW DOM was extracted from TW phytoplankton cultures maintained in the same condition as described above but amended with ^12^C sodium bicarbonate. Microbial remineralization experiments using ^12^C TW lysate_*PPL*_ extract with both surface and mesopelagic microbial assemblages were set up, sampled and analyzed following same protocols as described above. The relative abundance of 16S rDNA amplicons resolved across the various CsCl density fractions were compared between unamended control, ^12^C and ^13^C samples to see if a density shift of ^13^C incorporating taxa could be observed in ^13^C treatments compared to ^12^C or unamended control treatments (Supplementary Figure 3).

### SIP Experiment Data Analysis and Statistics

During the exponential growth phase of bacterioplankton, specific growth rates were calculated as the slope of the linear portion of the ln(BA) vs. time curve (Table 2). Stationary time points were determined from BA growth curve using growthcurver package in R and defined as 2 × t_*mid*_, where t_*mid*_ is the time when BA reached half carrying capacity assuming a logistic growth model (Sprouffske and Wagner, 2016). BC and DOC were interpolated at the stationary time points if sampling time point did not coincide with the model. Bacterial growth efficiency (BGE) was calculated as the ratio of the integrated area under BC curve with time to the integrated area of the DOC removal curve with time (Liu et al., 2020):

**TABLE 2:** Bacterial specific growth rate (μ, average ± SD), ∫ bacterial carbon (integrated and time normalized BC), ∫ DOC (integrated and time normalized DOC) and bacterial growth efficiency (BGE), BGE = ∫BCdt/∫DOCdt (Liu et al., 2020) over relevant time points.

Treatments	Exponential time (d)	μ (d^–1^)	Stationary time (d)	∫ BC (μmol C L^–1^)	∫ DOC (μmol C L^–1^)	BGE (%)
S control	0−0.5	0.49 ± 0.08	0.7 ± 0.1	0.01 ± 0.00	nr^a^	−
S TW lysate_*PPL*_	0−1.3	1.04 ± 0.01	1.4 ± 0.0	0.48 ± 0.02	2.1 ± 0.1	23
M control	0−1.0	0.88 ± 0.05	1.0 ± 0.0	0.05 ± 0.00	nr	−
M TW lysate_*PPL*_	0−1.0	2.44 ± 0.11	1.0 ± 0.0	0.85 ± 0.05	5.2 ± 0.6	16
M control	0−5.9	0.36 ± 0.04	4.9 ± 3.3	0.04 ± 0.03	nr	−
M Amino acid	1.4−3.4	1.03 ± 0.18	4.7 ± 0.1	0.33 ± 0.14	2.5 ± 0.8	13
M control	0−0.8	0.29 ± 0.14	No fit	−	−	−
M Syn lysate	0.6−2.2	1.56 ± 0.14	3.3 ± 0.1	0.16 ± 0.03	1.5 ± 0.9	14
M Syn lysate_*PPL*_	0.6−2.2	1.04 ± 0.37	2.5 ± 0.4	0.12 ± 0.05	nr	−
M Syn exudate_*PPL*_	1−2.5	1.17 ± 0.26	4.8 ± 2.6	0.08 ± 0.04	nr	−

*Abbreviations are the same as in Table 1. ^*a*^nr, not resolvable (<1 μmol C L^–1^).*

(4) $$\text{BGE}=\frac{\int_{\mathrm{T0}}^{\text{T}\,\text{stationary}}\mathrm{BC}\,\mathrm{dt}}{\int_{\text{T}\,0}^{\text{T}\,\text{stationary}}\mathrm{DOC}\,\mathrm{dt}}$$

Significant difference (*p* < 0.05) of BA curve between amendment treatments and control were tested through repeated measures ANOVA using Fit Model in JMP13 Pro. Difference of BA fold change, DOC removal and TDAA C removal among amendment treatments were evaluated with ANOVA and Kruskal-Wallis test (α = 0.05).

Bacterioplankton community structure of unfractionated samples at the family level was compared among treatments and time points. To avoid skewing by only a few of the most abundant taxa, 16S rRNA gene relative abundance was standardized as a z-score by each family taxon, calculated as the observed relative abundance of one taxon in a given sample minus the mean of relative abundance of the same taxon across all treatment samples and then divided by the standard deviation of relative abundance of that taxon across all treatment samples. This sets the relative abundance of each taxon to a standardized scale for comparison. Non-metric Multidimensional Scaling (NMDS) of bacterioplankton community structure of unfractionated samples was plotted based on Bray-Curtis dissimilarity using phyloseq, vegan and ggplot2 packages in R (Oksanen et al., 2007; Wickham, 2009; McMurdie and Holmes, 2013). Clusters in the NMDS plot were identified from hierarchical clustering using Simprof analysis (α = 0.05) (Supplementary Figure 4) from clustsig package in R (Whitaker and Christman, 2015).

To compare the ^13^C incorporation pattern of taxa among different substrates and determine how well each taxon was labeled, mean density was calculated for each ^13^C-incorporating taxon identified based on above criteria in each amended treatment as follows:

(5) $${\text{SA}}_{\text{ijk}}=\frac{{\text{RA}}_{\text{ijk}}}{{\text{RA}}_{\text{ijk[control]}}}$$

where standardized abundance (SA) equals the relative abundance (RA) of a taxon (i) in a given fraction (j) of amendment treatment (k) divided by that in the same density fraction of unamended control (k[control]). The standardized abundance in each fraction was then divided by the total standardized abundance of all fractions in that treatment as follows:

(6) $${\text{f}}_{\text{SAijk}}=\frac{{\text{SA}}_{\text{ijk}}}{\sum_{\text{j}}{\text{SA}}_{\text{ijk}}}$$

where f_SAijk_ is a metric of relative distribution (f) across all densities and ∑_j_ SA_ijk_ is the total standardized abundance of all fractions of a treatment for taxon i. This sets each taxon to a common scale (0−1). The mean density of each taxon (i) in each treatment (k) is calculated as:

(7) $$\text{mean}\,{\text{density}}_{\text{ik}}=\sum_{\text{j}}{\text{f}}_{\text{SAijk}}\times {\text{d}}_{\text{jk}}$$

where the metric of relative distribution is multiplied by the CsCl density of that fraction (d_*jk*_) and summed. To correct for CsCl density difference among treatments due to slight differences in the batches of CsCl buffer used, CsCl densities of whole unfractionated samples in different batches were normalized to the average of different batches for mean density comparison between treatments. The mean density for each taxon in each treatment was plotted as a heatmap with treatments and taxa names as *x* and *y* axis and the color scale representing the mean density value. Higher mean density values indicate that a taxon is more likely to have more ^13^C incorporated, and within the heatmap the taxa were clustered according to their mean density in different treatments.

### Field 16S rRNA Gene Sequencing and Comparison With SIP Data

To link SIP results to ambient bacterioplankton communities in the Sargasso Sea, field DNA samples were collected at the BATS site in three representative months before (January), during (April) and shortly after (June) convective mixing in 2017. 4 L of seawater at each discrete depth was filtered onto 0.2 μm Sterivex filters (polyethersulfone membrane, Millipore, Burlington, MA, United States) and stored in sucrose lysis buffer at −80°C. DNA was extracted using the phenol chloroform protocol (Giovannoni et al., 1996) and amplified with V1-V2 primer 27F (5′-AGAGTTTGATCNTGGCTCAG-3′) and 338RPL (5′-GCWGCCWCCCGTAGGWGT-3′) linked to “general” Illumina overhang adapters. Libraries were pooled in equimolar concentrations and sequenced using 2 × 250 Pair-End lanes with a MiSeq Reagent Kit v2 at the Center for Genome Research and Biocomputing (CGRB) at Oregon State University. Sequencing data were processed through the DADA2 pipeline as described above and relative abundance, assigned taxonomy, and sample metadata were compiled. Depth profiles of taxa at family level for top 300 m were compared (data are available at https://github.com/shutingliu/SIP_ms_FMICB_2020.git).

## Results

### Chemical Characteristics of Amended Substrates and Effects of PPL Extraction

The composition of DOM from Syn lysate after PPL extraction was markedly different compared to unaltered lysate. 2D NMR analysis was used to evaluate the effects of PPL extraction on major functional groups and chemical structures of phytoplankton derived DOM. While Syn lysate has more peaks in the bottom left quadrant (60−75 ppm in ^13^C chemical shift) of the ^13^C-^1^H HSQC plot, Syn lysate_*PPL*_ extract has more peaks in the top right quadrant (20−40 ppm in ^13^C chemical shift) (Figure 2), indicating different chemical characteristics between non-PPL and PPL extracts. Comparing our 2D NMR plots to a typical natural seawater high-molecular-weight (>1 kDa) DOM HSQC plot (Hertkorn et al., 2006) to infer the identity of the peaks, the bottom left quadrant in the Syn lysate plot corresponds to carbohydrate methylene and methine cross peaks and the top right quadrant in the Syn lysate_*PPL*_ plot corresponds to single bond C groups such as CH_2_ and CH_3_. TDAA C accounted for 15% of the amended Syn lysate DOC, while the TDAA C contribution of the Syn lysate after PPL extraction increased to 57% (Table 1). TDAA yield in the TW lysate_*PPL*_ extract (64%) was slightly higher than that in the Syn lysate_*PPL*_ extract. Urea concentrations in the Syn lysate, Syn lysate_*PPL*_ extract, and Syn exudate_*PPL*_ extract were 3.64, 0.08, and 7.29 μmol L^–1^, respectively.

### Changes in Bacterioplankton Abundance, DOC, and TDAA

**FIGURE 3:**
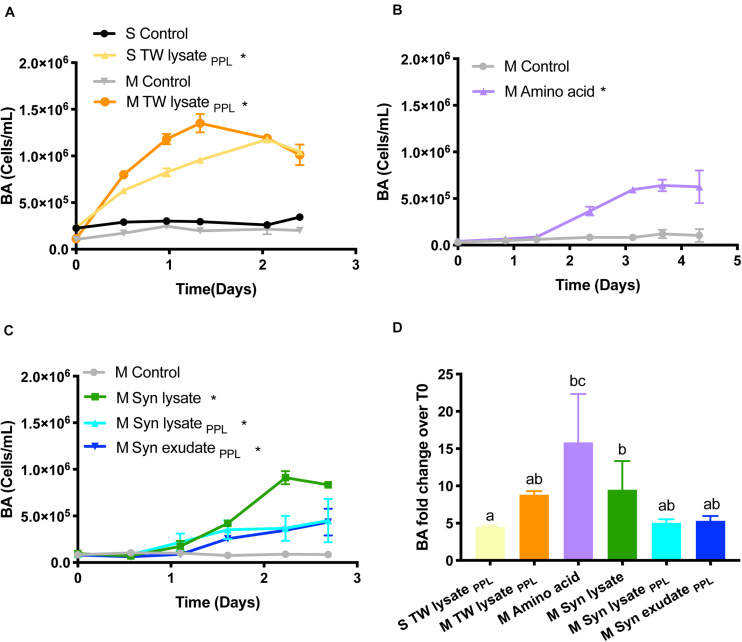
**(A–C)** Bacterioplankton abundance (BA) change with incubation time (* indicates significant difference compared to control). **(D)** BA fold change from T0 to the end (letters indicate significant difference).

Change in BA was significantly greater in each of the amendment treatments compared to control treatments (Figures 3A–C, repeated measures ANOVA, *p* < 0.05). The fold change in BA from T0 to TF was greatest in the amino acid treatment, followed by Syn lysate treatment and mesopelagic TW lysate_*PPL*_ treatment (Figure 3D). Comparing BA change among the mesopelagic Syn lysate, Syn lysate_*PPL*_, and Syn exudate_*PPL*_ treatments, bacterioplankton response in the lysate treatment was nearly twice of that in the PPL treatments (Figure 3C) and specific growth rate was greatest in the lysate treatment as well (Table 2).

**FIGURE 4:**
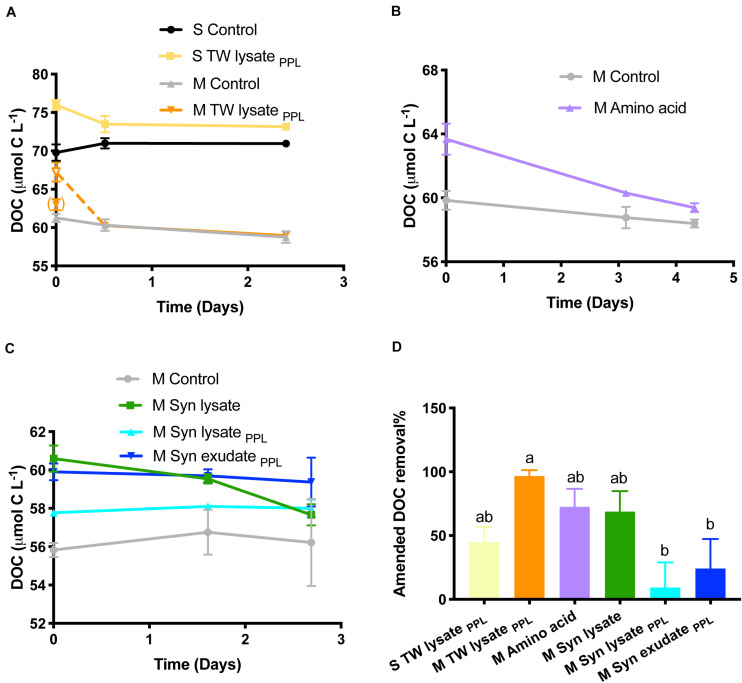
**(A–C)** DOC change with incubation time. DOC concentrations at T0 in the M TW lysate_*PPL*_ treatment (open symbol, connected with next data point in dashed line) were calculated based on measured TDAA C concentration and assuming a TDAA C: DOC ratio of 0.64 as measured for the amended substrate in the surface treatment (see text for details). Actual measured DOC at T0 in the M TW lysate_*PPL*_ treatment was in bracket as it was possibly underestimated due to sample preservation or storage problems. **(D)** Amended DOC removal percentage from T0 to the end (letters indicate significant difference).

**FIGURE 5:**
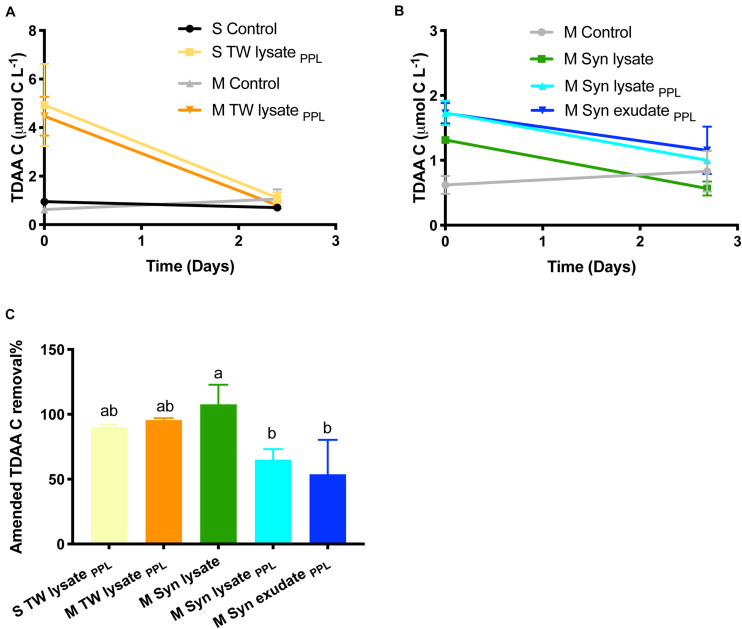
**(A,B)** TDAA C change in TW and Syn DOM treatments and their corresponding control treatments with incubation time. **(C)** Amended TDAA C removal percentage from T0 to the end (letters indicate significant difference).

The bulk DOC concentration measured at T0 of the surface TW lysate_*PPL*_ treatment met the expected enrichment of 6.2 μM C compared to the control (Figure 4A). However, for the mesopelagic TW lysate_*PPL*_ treatment the bulk DOC concentration of the T0 was much lower than the expected amended concentration (i.e., 1.7 μmol L^–1^ C above T0 control vs. the expected 6.2 μmol L^–1^ C). The TDAA C concentrations of the added TW lysate_*PPL*_ were similar between the surface and mesopelagic treatment (3.98 and 3.85 μmol L^–1^ C, respectively, see below Figure 5) indicating we did add the same amount of TDAA-C to each treatment. It is not clear why the bulk DOC yield was lower than expected in the mesopelagic treatment or lower than what was measured as added TDAA-C, but we suspect the bulk DOC sample was not preserved properly or was compromised in storage. To estimate the T0 DOC concentration for the mesopelagic treatment we used TDAA yield from surface treatment (i.e., amended TDAA-C was 64% of added DOC) to calculate expected T0 DOC in the mesopelagic TW lysate_*PPL*_ treatment (Table 1; see open symbol on Figure 4A). DOC drawdown was greatest in the three treatments that demonstrated the greatest bacterioplankton production i.e., amino acid, Syn lysate and mesopelagic TW lysate_*PPL*_ treatments (Figure 4 and Table 2). However, for the Syn DOM extracted with PPL cartridge, the DOC removal was minimal (<1 μmol C L^–1^), and the percent removal of DOC relative to amended concentrations was largely reduced (9−24%) in contrast to 69% removal of DOC in the non-PPL extracted Syn lysate treatment. BGE in treatments with resolvable DOC change (>1 μmol L^–1^ C) ranged from 13 to 23% (Table 2).

A major fraction of TDAA C was removed in all phytoplankton-derived DOM treatments compared to control within 3 days, suggesting the labile fraction of amended DOM was degraded rapidly (Figure 5). While >90% of amended TDAA C was removed in the TW lysate_*PPL*_ and Syn lysate treatments, a significantly lower percentage (54−65%) of amended TDAA C was remineralized in the Syn lysate_*PPL*_ and Syn exudate_*PPL*_ treatments.

### Unfractionated Microbial Community Structure

**FIGURE 6:**
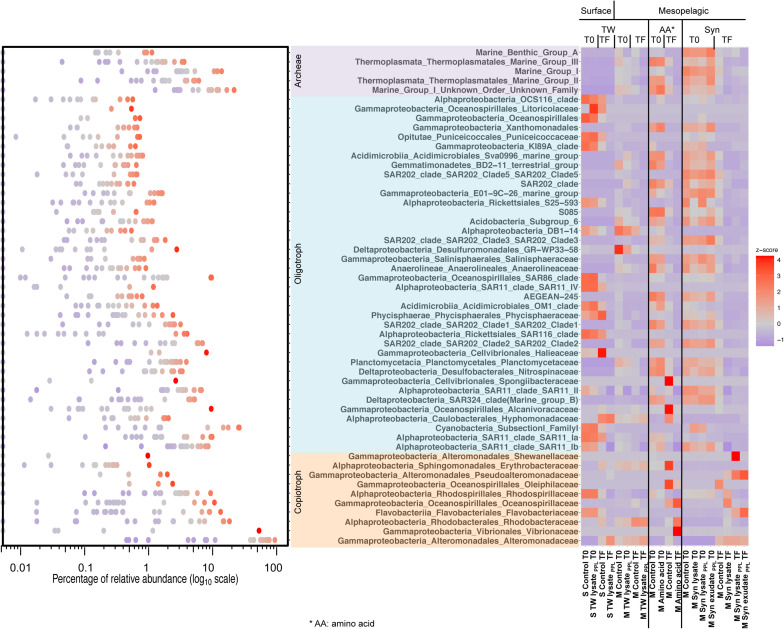
Left panel shows ranges of raw relative abundance data of each corresponding family taxon in the right panel. Right panel is a heatmap of z-score of major bacterioplankton family taxa (>0.5%) in every treatment at time point T0 and TF (2–4 days, see Table 1) of unfractionated samples. Z-score (standardized relative abundance by family taxa) was calculated as relative abundance of one taxon in the sample minus mean of relative abundance of the same taxon across all treatment samples and then divided by the standard deviation of relative abundance of that taxon across all treatment samples. This sets relative abundance of each taxon to a normalized scale to avoid skewing by only a few most abundant taxa like *Alteromonadaceae*. Higher z-score for each family taxon means relatively higher proportion of that taxon in the sample among all treatments.

Over the course of these experiments, copiotrophs became as the dominant members of the microbial communities. The T0 timepoint of the respective treatments represented the initial microbial community structure. 16S rRNA gene relative abundance data showed that the initial condition of the surface treatment was dominated by Cyanobacteria (25−26%), SAR11 clade (8% subclade Ib, 9−10% subclade Ia, 3−5% subclade II), *Flavobacteriaceae* (9%), *Rhodospirillaceae* (9%), and SAR86 clade (9−10%), whereas more members of the archaea marine group I (MGI) (9−22%), archaea MGII (2−6%), SAR202 clade (2−7%), and SAR324 clade (0.4−5%) comprised the initial condition of the mesopelagic treatments (Figure 6). Based on previous genomics and bioassay studies (Polz et al., 2006; Lauro et al., 2009; Buchan et al., 2014; Cárdenas et al., 2018), bacteria were pre-defined as copiotrophs or oligotrophs in our study. With incubation time, communities composed of diverse oligotrophs and archaea at T0 (accounting for an average relative abundance of 67%) all shifted to communities mostly dominated by copiotrophs at TF (accounting for an average relative abundance of 80%) in all treatments including unamended controls. *Alteromonadaceae* especially dominated in all treatments at TF (Figure 6, left panel), a common observation reported in other studies as possibly being due to bottle effects (Eilers et al., 2000; McCarren et al., 2010; Stewart et al., 2012; James et al., 2019). To avoid skewing of community structure by this dominant taxon, we standardized the relative abundance of each family (z-score; Figure 6, right panel). Comparing z-score of amended treatments with corresponding unamended control treatments at TF (Figure 6 and Table 3), most of the oligotrophs and archaea showed minimal or decreasing changes, except for the slight increase observed for members of the families *Hyphomonadaceae* and *Alphaproteobacteria* OCS116 clade in TW and/or Syn treatments compared to control. The greatest increase in z-scores was observed in copiotrophs (Table 3). The TF community structure was dominated by 53% *Vibronaceae*, 18% *Rhodobacteraceae*, and 11% *Oceanospirillaceae* in the amino acid treatment, and *Rhodobacteraceae*, *Oceanospirillaceae*, *Rhodospirillaceae* increased to 5%, 7%, and 6%, respectively in the Syn lysate treatment compared to unamended control. In contrast, more *Erythrobacteraceae* increased in the TW lysate_*PPL*_ treatment and more *Flavobacteriaceae* and *Pseudoalteromonadaceae* grew in the Syn_*PPL*_ treatments at TF.

**TABLE 3:** The z-score change of taxa (taxa with z-score change above ±1 standard deviation in any treatment were presented here, i.e., z-score change >1 or <−1) between DOM amendment treatment and control treatment at TF (2−4 days, see Table 1) in the unfractionated samples.

	Family Name	S TW lysate_*PPL*_	M TW lysate_*PPL*_	M Amino acid	M Syn lysate	M Syn lysate_*PPL*_	M Syn exudate_*PPL*_
Copiotroph	*Gammaproteobacteria_Alteromonadales_Alteromonadaceae*	**1.55**	0.51	–0.87	0.55	0.53	0.37
	*Gammaproteobacteria_Vibrionales_Vibrionaceae*	0.00	0.00	**4.48**	0.08	–0.02	–0.02
	*Alphaproteobacteria_Rhodobacterales_Rhodobacteraceae*	**−1.08**	–0.07	**2.45**	0.82	0.23	0.00
	*Flavobacteriia_Flavobacteriales_Flavobacteriaceae*	–0.85	0.25	–0.08	0.34	**1.71**	**3.38**
	*Gammaproteobacteria_Oceanospirillales_Oceanospirillaceae*	0.00	–0.60	**1.88**	**2.43**	0.01	–0.13
	*Alphaproteobacteria_Rhodospirillales_Rhodospirillaceae*	**−1.13**	–0.11	–0.32	**1.54**	–0.26	–0.39
	*Gammaproteobacteria_Oceanospirillales_Oleiphilaceae*	0.00	0.00	**−3.10**	**−2.34**	**−2.34**	**−2.34**
	*Gammaproteobacteria_Alteromonadales_Pseudoalteromonadaceae*	0.32	0.00	0.00	0.90	**2.82**	**3.61**
	*Alphaproteobacteria_Sphingomonadales_Erythrobacteraceae*	–0.76	0.46	**−4.30**	0.00	0.00	0.13
	*Gammaproteobacteria_Alteromonadales_Shewanellaceae*	0.00	0.00	0.00	0.00	**4.48**	0.00
Oligotroph	*Planctomycetacia_Planctomycetales_Planctomycetaceae*	0.00	–0.58	–0.84	**−1.33**	**−1.07**	**−1.04**
	*Alphaproteobacteria_*SAR11_clade_SAR11_Ib	**−1.11**	–0.61	–0.75	**−1.31**	–0.56	–0.62
	*Alphaproteobacteria_*SAR11_clade_SAR11_Ia	**−2.24**	–0.14	–0.43	–0.18	–0.18	–0.18
	*Alphaproteobacteria_Caulobacterales_Hyphomonadaceae*	0.31	0.36	**−2.19**	0.00	0.00	0.31
	*Gammaproteobacteria_Oceanospirillales_Alcanivoracaceae*	–0.49	–0.41	**−4.31**	–0.36	–0.36	–0.34
	*Alphaproteobacteria_*SAR11_clade_SAR11_II	–0.79	–0.76	**−1.48**	**−1.47**	–0.74	–0.75
	*Gammaproteobacteria_Cellvibrionales_Spongiibacteraceae*	0.00	0.00	**−4.27**	–0.05	–0.05	–0.05
	*Deltaproteobacteria_Desulfobacterales_Nitrospinaceae*	0.00	–0.58	**−1.06**	**−1.01**	–0.77	**−1.12**
	*Gammaproteobacteria_Cellvibrionales_Halieaceae*	**−4.44**	0.00	0.00	0.00	0.00	0.00
	*Alphaproteobacteria_Rickettsiales_*SAR116_clade	**−2.03**	0.00	0.00	0.00	0.00	0.01
	*Phycisphaerae_Phycisphaerales_Phycisphaeraceae*	**−3.76**	–0.32	–0.48	–0.51	–0.51	–0.33
	*Acidimicrobiia_Acidimicrobiales_*OM1_clade	**−1.18**	0.00	–0.69	–0.28	–0.28	–0.28
	*Alphaproteobacteria_*SAR11_clade_SAR11_IV	**−1.35**	0.00	0.00	–0.04	–0.04	0.00
	*Alphaproteobacteria_*DB1-14	–0.65	**−1.24**	0.00	0.00	0.00	0.00
	*Acidobacteria_*Subgroup_6	0.00	–0.64	–0.33	**−1.18**	–0.74	–0.99
	SAR202_clade	0.00	–0.45	**−1.37**	–0.50	–0.89	–0.89
	SAR202_clade_SAR202_Clade5_SAR202_Clade5	0.00	0.00	0.00	–0.98	**−1.19**	**−1.19**
	*Opitutae_Puniceicoccales_Puniceicoccaceae*	**−1.49**	0.00	0.00	0.00	0.00	0.00
	*Gammaproteobacteria_Oceanospirillales_Litoricolaceae*	**−2.00**	0.00	0.00	0.00	0.00	0.00
	*Alphaproteobacteria_*OCS116_clade	**−2.14**	0.00	0.00	0.10	0.00	0.18
Archaea	Marine_Group_I_Unknown_Order_Unknown_Family	0.00	–0.58	**−1.11**	–0.61	–0.35	–0.30
	*Thermoplasmata_Thermoplasmatales_*Marine_Group_III	0.00	–0.59	**−1.03**	–0.81	–0.68	–0.79

*Positive z-score change means relative abundance of taxa was higher in amendments compared to control at the end of incubation, and negative z-score change means the opposite. The z-score change >1 was bolded and underscored, and <−1 was bolded. Treatment abbreviations are the same as in Table 1.*

**FIGURE 7:**
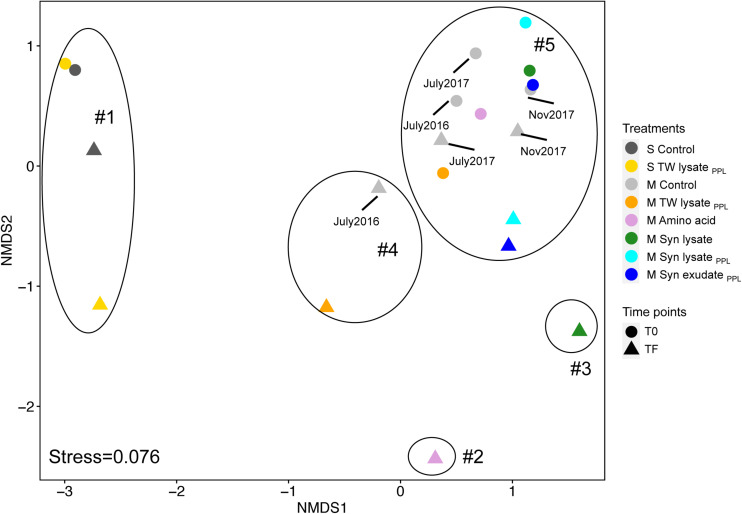
Non-metric Multidimensional Scaling plot of bacterioplankton community structure of unfractionated samples at T0 and TF (final DNA harvesting time point) based on Bray-Curtis distance (*Alteromonadaceae* were excluded to avoid bias by its dominance at TF samples). Five clusters were grouped based on hierarchical clustering analysis and simprof analysis (α = 0.05) at threshold of 70% dissimilarity (identified as in Supplementary Figure 4).

To evaluate the response of other taxa, *Alteromonadaceae* lineage identified as non-^13^C-incorporating taxon (more discussion below) were excluded for NMDS and clustering analysis as the dominance of *Alteromonadaceae* in all TF samples reduced proportions of all other taxa as shown in Figure 6. Microbial community structure of whole unfractionated samples formed distinct clusters on the NMDS plot (Figure 7 and Supplementary Figure 4). Surface communities (cluster 1) were separated from mesopelagic communities on the NMDS1 axis, and incubation and treatment effects were separated on the NMDS2 axis (Figure 7). While bacterioplankton community observed at TF in the amino acid and Syn lysate treatment (cluster 2 and 3, respectively) separated furthest from all other mesopelagic samples, corresponding PPL DOM amendments demonstrated TF community structure that was grouped with unamended control samples (Figure 7, cluster 4 and 5), illustrating a smaller change in community structure in response to PPL-extracted DOM.

### Identifying Microbial Taxa Incorporating ^13^C

**FIGURE 8:**
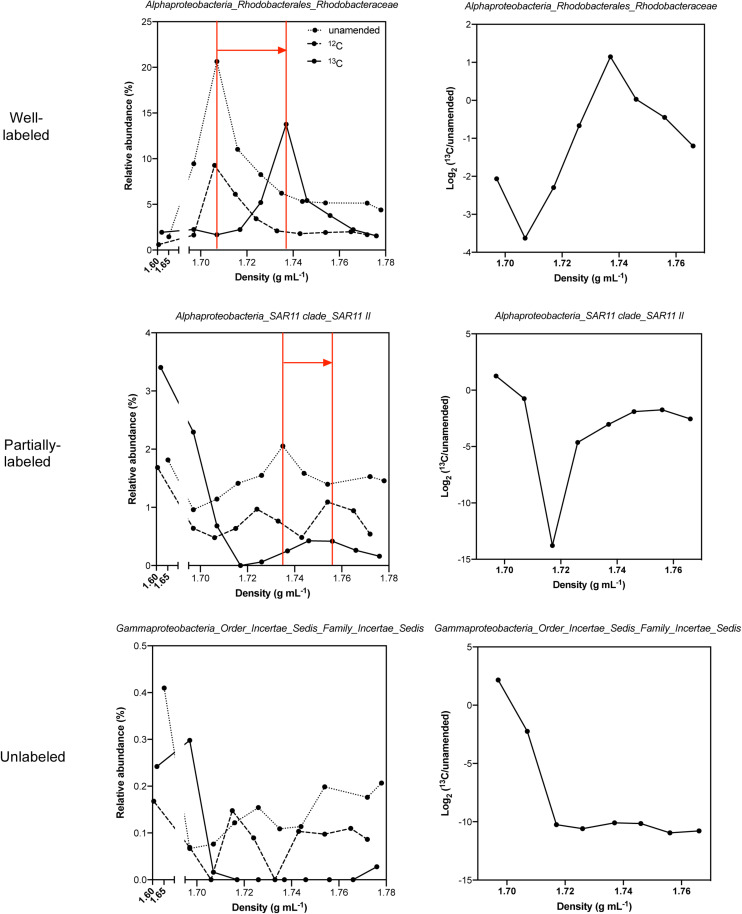
Method validation of identifying ^13^C-incorporating family taxa. Relative abundance vs. CsCl density and unamended control normalized and log2 transformed relative abundance vs. CsCl density plots for examples of well-labeled, partially labeled and unlabeled taxa among ^13^C-TW lysate_*PPL*_, ^12^C-TW lysate_*PPL*_, and unamended control treatments. Red lines and arrows indicated shift to heavier density in the ^13^C treatment compared to the ^12^C and unamended control treatments. Since lightest and heaviest fractions are mixed with water or CsCl, DNA quantities were low and densities deviated from normal range (as shown in left panel x break axis), thus these two fractions were excluded in following analyses.

One of the challenges with DNA-SIP is designing an approach that clearly identifies taxa that incorporated ^13^C from those taxa that do not. Ideally, taxa that incorporate ^13^C increase their DNA’s density and can thereby be separated into distinct bands along a CsCl density gradient from those organisms that do not incorporate the “heavy” label. However, when studying a mixed population of bacterioplankton, both the variable GC content of the organisms’ DNA and the maintenance of unlabeled cells in initially abundant populations can lead to smearing of DNA along a CsCl gradient (Nelson and Carlson, 2012). For DNA-SIP, comparing the relative abundance profiles of specific taxon along a CsCl density gradient grown on ^13^C DOM to that in the corresponding ^12^C DOM incubations is a direct way to identify taxa with clear density shift resulting from ^13^C-incorporation (Figure 8, complete comparison for all labeled taxa in that experiment shown in Supplementary Figure 3).

Distinctive patterns based on labeling efficiency emerge from the comparisons of labeled treatments versus unlabeled controls. For well-labeled taxa, such as *Rhodobacteraceae*, *Hyphomonadaceae*, *Erythrobacteraceae*, and *Bdellovibrionaceae* in the TW lysate_*PPL*_ treatments, their relative abundance maxima demonstrated a clear shift to heavier density by 0.020−0.031 g mL^–1^. For taxa, such as SAR11 II and *Acidimicrobiales* OM1 clade, whose ^12^C profiles revealed relative abundance profiles high in the light fractions and uniformly distributed across several other density fractions or enriched at certain medium density fractions, the ^13^C profiles shifted to ones that were “V” shaped across density fractions i.e., enriched in both the light and heavy densities with a dip at the medium density fractions. For unlabeled taxa, relative abundance was highest in light fractions and then decreased with increasing densities. The relative abundance patterns of corresponding taxa were similar between the unamended control and the ^12^C treatment and were well separated from the taxa/density profile of ^13^C-incorporating organisms. Therefore, while logistical constraints limited the number of simultaneous ^12^C incubations, we were able to use the taxa/density profiles of the unamended controls as a valid approach for differentiating microbial taxa incorporating ^13^C from those with natural GC content variation. For instance, *Alteromonadaceae* is an example of a false positive in the taxa/density profile that was corrected after normalization to unamended control (Supplementary Figure 5). Although *Alteromonadaceae* was the most abundant taxon after incubation in many of the treatments and its DNA often smeared to multiple density fractions, it did not show enrichment pattern in the heavy density fractions after normalizing its relative abundance in the ^13^C treatment by that of the unamended control (Supplementary Figure 5). Thus, we excluded *Alteromonadaceae* as ^13^C-incorporating taxon in our study, consistent with the observation that this family increased relative abundance in the control treatments without DOM amendment. A complete dataset in every treatment for identification of ^13^C-incorporating taxa using taxa/density profiles before and after normalization to unamended control is shown in Supplementary Figures 1, 2.

Although the overall unfractionated microbial community structure revealed enrichment of copiotrophs with minimal changes of oligotrophs and archaea in the amendment treatments (Figure 6), the more sensitive DNA-SIP approach clearly identified the response of some oligotrophs and archaea to amended ^13^C DOM substrates (Table 4). The simple compound mixture of ^13^C amino acids were largely incorporated by a variety of copiotrophic bacterioplankton, including *Vibrionaceae*, *Oleiphilaceae*, *Pseudoalteromonadaceae*, *Rhodobacteracea*, and *Eryththrobacteraceae*. However, the ^13^C culture lysate and exudate composed of a complex mixture of compounds were incorporated by both copiotrophs and oligotrophs. The responding oligotrophs included taxa such as members of the SAR86, *Salinisphaerales*, *Rickettsiales*, SAR11, SAR202, *Acidimicrobiales*, and *Anaerolineaceae* clades. Of the subset of taxa presented in Table 4, only oligotrophs were detected as ^13^C incorporators in treatments where the Syn lysate or exudate was further treated by extraction with PPL cartridges, whereas both copiotrophs and oligotrophs incorporated ^13^C untreated Syn lysate. Marine benthic group A archaea, which are chemolithoautotrophs, also became labeled in the Syn exudate_*PPL*_ treatment.

**TABLE 4:** Taxa at the family level incorporating ^13^C (x) in amended treatments.

	Family name	S TW lysate_*PPL*_	M TW lysate_*PPL*_	M Amino acid	M Syn lysate	M Syn lysate_*PPL*_	M Syn exudate_*PPL*_
Copiotroph	*Gammaproteobacteria_Vibrionales_Vibrionaceae*			x			
	*Gammaproteobacteria_Oceanospirillales_Oleiphilaceae*			x			
	*Gammaproteobacteria_Alteromonadales_Pseudoalteromonadaceae*			x	x		
	*Alphaproteobacteria_Rhodobacterales_Rhodobaceraceae*	x		x			
	*Alphaproteobacteria_Sphingoonadales_Erythrobacteraceae*	x	x	x			
Oligotroph	*Plantomycetacia_Plantomycetales_Plantomycetaceae*			x			
	*Gammaproteobacteria_Oceanospirillales_*SAR86 clade				x	x	x
	*Gammaproteobacteria_Thiotrichales_Piscirickettsiaceae*			x			
	*Gammaproteobacteria_Salinisphaerales_Salinisphaerales*						x
	*Alphaproteobacteria_Caulobacterales_Hyphomonadaceae*	x					
	*Alphaproteobacteria_Rickettsiales_*S25-593				x	x	x
	*Alphaproteobacteria_*SAR11 clade_SAR11 II	x					
	*Deltaproteobacteria_*SAR324 clade (Marine group B)						x
	*Deltaproteobacteria_Desulfobacterales_Nitrospinaceae*				x		
	*Deltaproteobacteria_Bdellovibrionales_Bdellovibrionaceae*		x				
	SAR202 clade_SAR202 Clade1_SAR202 Clade1						x
	SAR202 clade_SAR202 Clade2_SAR202 Clade2				x	x	x
	SAR202 clade_SAR202 Clade3_SAR202 Clade3					x	
	*Acidimicrobiia_Acidimicrobiales_*OM1 clade	x		x			
	*Acidimicrobiia_Acidimicrobiales_*Sva0996 marine group						x
	*Anaerolineae_Anaerolineales_Anaerolineaceae*					x	x
	*Acidobacteria_*Subgroup 6				x		
Archaea	*Marine Benthic group A*						x

*Treatment abbreviations are the same as in Table 1.*

### Mean Density

**FIGURE 9:**
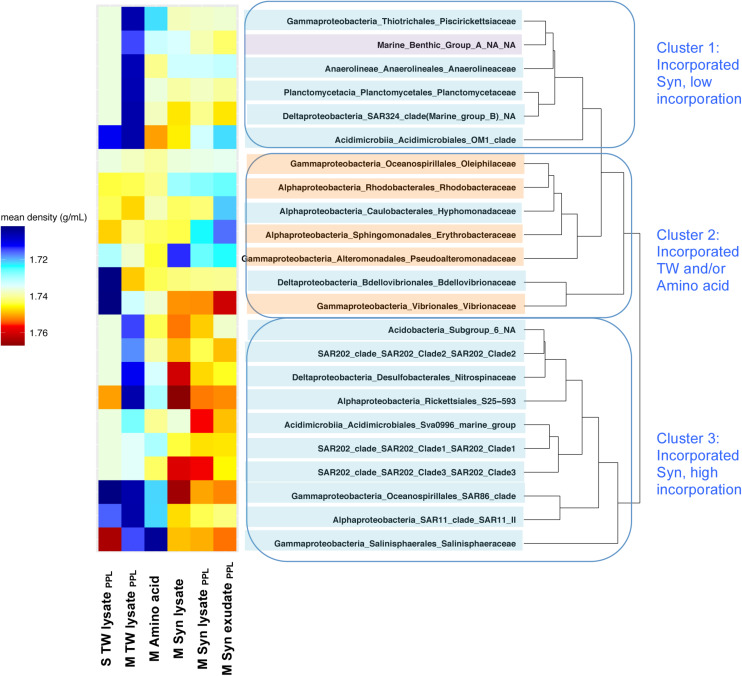
Mean CsCl density where each bacterioplankton taxon in Table 4 was resolved at TF among treatments. The shading on the taxa represents the following: orange-shaded, copiotroph; blue-shaded, oligotroph; purple-shaded, archaea. Warm colors represent the mean CsCl density associated with taxon that likely incorporated more ^13^C in that treatment.

After identifying taxa that incorporated ^13^C-compounds, the mean density (a metric of how well the population is labeled) of each ^13^C-incorporating taxon was calculated across all treatments resulting in three clusters of microbial taxa. Cluster 1 represents a group with low incorporation of Syn DOM; cluster 2 represents a group with relatively high incorporation of DOM derived from TW and/or amino acid; cluster 3 represents a group with relatively high incorporation of DOM derived from Syn (Figure 9). The heatmap in Figure 9 indicated that greater mean densities were resolved for cluster 3 compared to cluster 1 indicating that taxa in cluster 3 were capable incorporating more ^13^C Syn-derived DOM and thus had more labeled cells in the respective treatments. Cluster 2 was dominated by copiotrophs, cluster 1 was a mixture of oligotrophs and archaea, and cluster 3 was exclusively comprised of oligotrophs. Comparing each taxon across rows reveals whether a microbial taxon had the capability of incorporating multiple substrates (i.e., generalists). For instance, some organisms in cluster 2 were able to incorporate not only amino acids, but also TW-derived DOM, suggesting they are generalists; whereas most organisms in cluster 1 and cluster 3 seem to be specialized in utilizing Syn DOM.

### Field Bacterioplankton Communities in the Sargasso Sea

**FIGURE 10:**
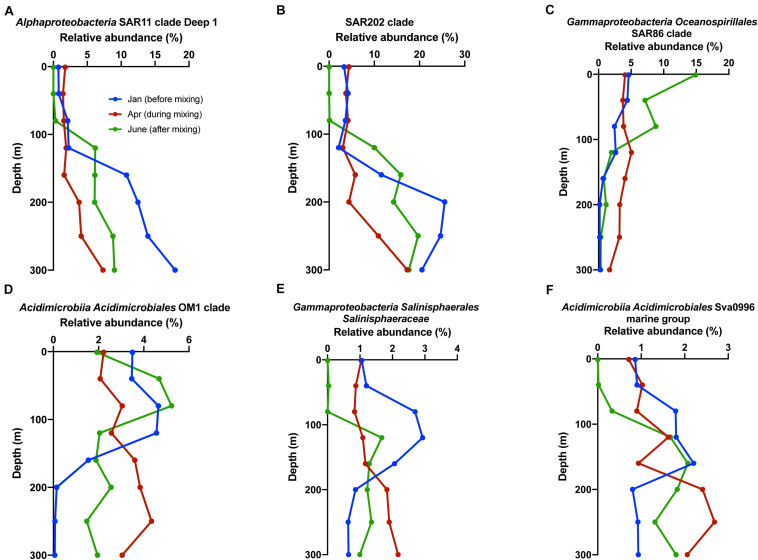
Relative abundance at family level of members in panels **(A)** SAR11 clade, **(B)** SAR202 clade, **(C)** SAR86 clade, **(D)** *Acidimicrobiales* OM1 clade, **(E)** *Salinisphaeraceae*, and **(F)** *Acidimicrobiales* Sva0996 clade in top 300 m at BATS station in the Sargasso Sea before (January), during (April), and shortly after (June) convective mixing event in 2017.

To examine if the oligotrophs showing ^13^C incorporation in our SIP study were also present in the natural oligotrophic seawater of Sargasso Sea, the relative abundance of oligotrophs in ambient seawater at BATS was compared between 3 months coinciding with the time before, during, and shortly after deep convective mixing (Figure 10). Some representative family taxa showed growth in the mesopelagic zone during or shortly after winter convective mixing, which exported semi-labile and semi-refractory DOM, including springtime accumulated phytoplankton-derived DOM, from surface to mesopelagic. Members of SAR11 deep clade, SAR202, SAR86, *Acidimicrobiales* OM1, *Acidimicrobiales* Sva0996, and *Salinisphaeraceae*, which showed a response to convective mixing, were also found to incorporate phytoplankton PPL DOM in the SIP results (Table 4), suggesting a potential link between microbial taxa responding in SIP incubations with PPL-altered DOM and the *in situ* response of those oligotrophs in natural seawater of Sargasso Sea.

## Discussion

### Change of DOM Quality via PPL

The limited bacterioplankton production and DOC removal observed in the Syn lysate _*PPL*_ compared to the unaltered Syn lysate treatments (Figures 3, 4) indicates that PPL extraction removed a large fraction of the most bioavailable compounds but appeared to retain a more recalcitrant fraction of DOM. The PPL resin, a functionalized styrene-divinylbenzene, extracts a wide range of molecules ranging from highly polar to non-polar and relatively enriches low-molecular weight compounds and carboxyl-rich alicyclic molecules (CRAM) that are a major fraction of refractory DOM (Hertkorn et al., 2006; Dittmar et al., 2008; Bayer et al., 2019). Fluorescence and freshness indices based on optical properties decreased in PPL-extracted DOM (Wünsch et al., 2018) also suggest PPL enriches recalcitrant DOM. Consistent with the present study, Hansen et al. (2019) showed that PPL-extracted DOM from hydrothermal vents was resistant to microbial degradation compared to bulk DOM from the same site.

The extraction efficiency of PPL on phytoplankton-derived DOM observed in the present study (8−15%) was lower than that reported for bulk marine DOM (43−65%) (Dittmar et al., 2008). Since compounds vary in their retention on PPL cartridges from 0 to 100% (Johnson et al., 2017), the lower extraction efficiencies we observed could be attributed to organic composition matrix effects. For example, carbohydrates tend to have low retention efficiencies on PPL cartridges (Swenson et al., 2014; Raeke et al., 2016). PPL adsorption and extraction rely on reverse phase chemistry that mostly favors non-polar compounds. The low pH (pH ∼3) necessary to charge the PPL cartridges can protonate proteins and peptides (pKa 4-10) and thus reduce the extraction efficiency of proteins and peptides (Stücheli et al., 2018). Carbohydrates and proteins account for 5−50% and 25−50% of marine phytoplankton biota, respectively (Emerson and Hedges, 2008). The wide variety of chemical mechanisms that can result in DOM loss during PPL extraction suggests that low efficiency of DOM recovery from phytoplankton lysates is the result of some DOM compounds passing through the PPL cartridges. Consistent with previous studies, PPL extraction efficiencies for DOM derived from phytoplankton cultures are typically lower, ranging from 2% to 50%, and culture DOM is usually more saturated, less oxygenated, and contains more heteroatoms in comparison to seawater DOM (Lechtenfeld et al., 2015; Wienhausen et al., 2017).

Among the substrates used in this study, amino acids represented model labile compounds while phytoplankton-derived DOM represented a complex combination of compounds classes such as carbohydrates, proteins, and lipids (Grossart et al., 2006). For example, polysaccharide laminarin was found to contribute to 26 ± 17% of microalgae-derived POC and diatoms drove laminarin production during spring blooms (Becker et al., 2020). Cyanobacteria possess polyphosphate as a common phosphorus storage compound, cyanophycin as a nitrogen storage product, and glycogen as a storage compound of both carbon and energy (Kromkamp, 1987; Martin et al., 2014). Cyanobacteria have also been widely used as a resource for biofuel lipid production (Farrokh et al., 2019). The greater change of BA in the amino acid treatment compared to others (Figure 3) is consistent with the explanation that simple amino acids may fuel anabolism of bacterioplankton biomass compared to substrates like complex carbohydrate and lipid components that might be more catabolized as an energy source. However, these chemical characteristics of phytoplankton DOM substrates were altered by PPL extraction. The NMR 2D HSQC plot (Figure 2) reveals that Syn lysate was enriched in carbohydrate methylene and methine cross peaks in the bottom left quadrant of plot, while Syn lysate extracted by the PPL cartridge was enriched in methyl, methylene and methine cross peaks (Hertkorn et al., 2006). These patterns in functional groups suggest an inefficient retention of labile carbohydrate or carbohydrate-like DOM by PPL extraction while retaining aliphatics more efficiently because of their hydrophobic properties. Perminova et al. (2014) demonstrated PPL extraction of Arctic DOM contained high percentage (35−43%) of aliphatic alkane protons. The single bond resonance retained on the PPL extract is consistent with the enriched methyl proton resonance of intact and oxidized carotenoids that are widespread in phytoplankton and likely to be the precursors of refractory DOM in the seawater (Repeta and Gagosian, 1982; Arakawa et al., 2017). It is intriguing that TDAA yield was greater in PPL extracts compared to unaltered phytoplankton DOM (Table 1). Consistent with the studies above, the NMR analyses in the present study indicated that carbohydrates were not well retained on PPL (Figure 2), as such the lower retention of carbohydrates on PPL relative to that of amino acids or amino acid polymers led to an enriched TDAA percentage in the PPL extracts. We hypothesize that enriched TDAA in PPL extracts were more likely to be hydrolyzable amino acids that are less bioavailable, such as amino acids bound to humic substances or D-amino acids from cell-wall biopolymers, rather than labile amino acids from proteins or peptides (Repeta, 2015). Evidence in support of this hypothesis includes: (1) the mismatch between the low extraction efficiency (8−15%) of phytoplankton-derived DOM through PPL and typical 25−50% of phytoplankton biomass as proteins suggested proteins were not well retained on PPL; (2) the percent of TDAA removed in the Syn PPL extracts was lower compared to unaltered Syn DOM during the short incubation (Figure 5), indicating TDAA in Syn PPL extracts were less labile. Overall, our bioassay experiment and NMR analysis showed that PPL pretreatment on DOM served as an approach to yield DOM of differing quality, however, further studies on the actual effect of PPL on phytoplankton-derived DOM compounds or molecular specific alterations through techniques like ultrahigh resolution mass spectrometry are needed.

### DOM Quality Shapes Microbial Community Structure

16S rRNA gene relative abundance after standardization (z-score) and NMDS plot revealed that PPL extracted substrates shaped copiotrophs in microbial community structure distinctly from the amino acid and Syn lysate where no PPL pretreatment was performed (Figures 6, 7). Copiotrophs such as *Vibrionaceae*, *Rhodobacteraceae* (of which *Roseobacter* clade belongs to), and *Oceanospirillaceae*, that typically grow well in the presence of labile DOM (Eilers et al., 2000; Romera-Castillo et al., 2011; Landa et al., 2016; Goldberg et al., 2017) were the dominant responding lineages in the amino acid and Syn lysate treatments of the present study. In contrast, *Flavobacteriaceae*, *Pseudoalteromonadaceae*, and *Erythrobacteraceae* showed the greatest change in relative abundance in the PPL-altered DOM (TW_*PPL*_ or Syn_*PPL*_) treatments compared to unamended control treatments. Members of *Flavobacteria* are adapted to use HMW compounds and *Pseudoalteromonas* and *Erythrobacter* are capable of producing extracellular enzymes to hydrolyze polymers (Cottrell and Kirchman, 2000; Teeling et al., 2012; Cárdenas et al., 2018; Zhang et al., 2018), suggesting they may be involved in breaking down complex molecular structures in the PPL DOM treatments.

Microbial community structure based on the relative abundance of 16S rDNA amplicons is effective for measuring changes caused by cell growth. However, relative abundance analyses can overrepresent some copiotrophs because their ability to rapidly upshift growth rates and the fact that some have multiple 16S rRNA gene copies per cell that obfuscate the slow-growing oligotrophs (López-Pérez et al., 2012; Větrovský and Baldrian, 2013). Because relative contribution of a taxon is not necessarily correlated to absolute change in biomass, one can observe a decrease in the relative abundance of a slow-growing taxon if fast-growing taxa or taxa with multiple gene copies per cell outpace production of the oligotroph. In the present study, we used relatively short incubations (2−4 days) to limit “cross-feeding” effects, i.e., the incorporation of labeled secondary metabolites excreted by other organisms, which limited the ability to resolve slow-growing taxa in an analysis of total unfractionated community structure. As expected, minimal or negative changes of oligotroph relative abundances between amended and unamended treatments at TF were observed (Figure 6 and Table 3) and this is precisely why the sensitive DNA-SIP approach was used to resolve which taxa were incorporating ^13^C labeled compound into their biomass. When directly tracking the ^13^C incorporation into biomass through DNA-SIP, more changes in slow-growing oligotrophs could be sensitively resolved compared to relying on shifts in amplicon abundance (community structure) in an unfractionated DNA sample. This allowed us to identify specific microbial lineages capable of responding to DOM sources of varying quality (see discussion below).

### Factors to Consider When Identifying SIP ^13^C Labeling Patterns

Both ^13^C well-labeled and partially labeled microbial taxa were identified as ^13^C-incorporting microbes (Figure 8 and Supplementary Figures 1, 2). When the relative abundance of one taxon increases with CsCl density and is primarily enriched in heavy fractions, it is clearly well-labeled with heavy isotope in the biomass. Partially labeled patterns, on the other hand, can be due to the high abundance of certain microbial taxa at the beginning of incubation and/or slow-growth of a taxon resulting in only a portion of that taxon’s population becoming labeled over the course of the incubation (Nelson and Carlson, 2012). For example, SAR11, which comprises a major fraction of the ambient community (20−30%) in oligotrophic seawater, exhibits a V-shape relative abundance vs. density profile (Figure 8). The short incubation time is not long enough to replace all ^12^C DNA component of the SAR11 population with ^13^C in the slow-growing population (Nelson and Carlson, 2012), resulting in the bimodal distribution of DNA across density fractions.

Most SIP studies amended with model compounds use a ^12^C treatment to compare with the ^13^C amendment for identifying ^13^C incorporators. In our validation test, the density shift between our ^13^C treatments and ^12^C treatments (0.020−0.031 g mL^–1^) was larger than 0.01 g mL^–1^ (Figure 8 and Supplementary Figure 3), equating to ∼ 28% of ^13^C incorporation which is more than the minimum percentage (20%) typically required for separating ^13^C and unlabeled organisms (Uhlik et al., 2009). Logistically, it is not feasible to maintain the same growth rate of different batches of phytoplankton cultures and harvest the phytoplankton-derived DOM of the exact same quality; thus, we could not include a ^12^C DOM amendment for every experimental treatment. However, the similarity of bacterioplankton taxa/density profiles across density fractions observed between the ^12^C DOM treatment and unamended control treatment validates our approach of using unamended control to correct for false positive signals when assessing the relative abundance patterns of taxa across density fractions (Figure 8 and Supplementary Figures 3, 5).

### DOM Quality Determined Different ^13^C-Incorporating Microbial Taxa

^13^C-incorporating microbes were identified via DNA-SIP fractionation method. While amino acids were incorporated mostly by copiotrophs, PPL DOM substrates enriched for a variety of oligotrophs (Figure 9 and Table 4). Extensive studies have shown the correlation between DOM with varying reactivity and distinct microbial community structures (Covert and Moran, 2001; Nelson et al., 2013; Landa et al., 2016; Guerrero-Feijoo et al., 2017; Quigley et al., 2019), implying that DOM quality is an important bottom-up driver of the responding microbial community structure and resource partitioning among different microbial groups. This DNA-SIP study directly linked different sources and pretreatments of amended DOM with specific microbial taxa capable of incorporating the labeled DOM. Not surprisingly, the present study demonstrates that PPL treatment of DOM derived from different phytoplankton sources yielded DOM of differing quality and bioavailability. Thus, the resulting bacterioplankton capable of incorporating the ^13^C labeled compounds differed between phytoplankton-derived DOM sources. TW lysate_*PPL*_ extracts were more bioavailable (Figure 4D) and demonstrated a greater number of copiotrophs capable of incorporating TW lysate_*PPL*_ than Syn lysate_*PPL*_ extracts. Differences between diatom- (*Phaeodactylum*) and cyanobacteria- (*Synechococcus*) derived DOM have also been revealed in a previous study (Landa et al., 2014), showing that diatom DOM contained a greater percentage of labile amino acids and neutral sugars in the total DOC than cyanobacteria DOM. Higher TDAA yield and percent removal in the TW lysate_*PPL*_ compared to Syn lysate_*PPL*_ treatment (Figure 5 and Table 1) supports the notion of varying DOM quality originating from differing phytoplankton sources.

The present study demonstrated the dominant role of various copiotrophs capable of incorporating ^13^C labeled components of labile amino acids and Syn lysate (Table 4). These results are consistent with previous incubation studies that show copiotrophs (i.e., *Rhodobacteraceae*, and *Vibrionaceae)* respond to or incorporate amended labile phytoplankton-derived DOM and amino acid mixtures (Cottrell and Kirchman, 2000; Eilers et al., 2000; Pinhassi and Berman, 2003; Morris et al., 2006; Mayali et al., 2012; Dadaglio et al., 2019). The importance of members of the *Alphaproteobacteria* and *Gammaproteobacteria* copiotrophs in incorporating labile DOM is supported by transcriptomic and proteomic studies demonstrating high abundance of amino acid, peptide, and carbohydrate transporters expressed by those bacterioplankton in the water column, which can account for ∼30−85% of total protein expression from euphotic to bathypelagic zones (Poretsky et al., 2010; Bergauer et al., 2018). Members of *Alphaproteobacteria* and *Gammaproteobacteria* possess polyphosphate kinases or genes encoding polyphosphate kinase and cyanophytinases (Tang et al., 2012; Achbergerová and Nahálka, 2014; Jones et al., 2016), and *Pseudoalteromonas* displayed beta-glucanase activity (Nakatani et al., 2010), which might facilitate their utilization of phytoplankton lysate. It is noted that a few copiotrophs like *Flavobacteriaceae* and *Alteromonadaceae* showed growth in the unfractionated community structure (Figure 6) but were not identified as ^13^C incorporators in the PPL DOM treatments (Table 4). As our PPL-altered DOM substrates were not 100% ^13^C labeled, it is possible that a few taxa responded to the unlabeled components in the PPL extracts or unlabeled secondary metabolites generated during incubations.

Many of the oligotrophs that did not account for a high relative abundance in the unfractionated samples, presumably due to their limited growth rate, were identified as ^13^C incorporators in our PPL DOM and Syn lysate treatments after fractionation of DNA across CsCl density gradient. This discrepancy between the analyses of unfractionated community structure and DNA-SIP identified DOM incorporators highlights the advantage of using the sensitive SIP technique to identify response of oligotrophs to varying DOM sources and quality. Syn lysate is a mixture of abundant labile and less abundant recalcitrant DOM (Zheng et al., 2019a), therefore ^13^C incorporation by both copiotrophs and oligotrophs was possible. For example, members of the *Acidobacteria* clade, a responding lineage to the Syn lysate treatment has been shown to take up cellulose, a byproduct of cyanobacteria production (Nobles et al., 2001; Eichorst and Kuske, 2012; Zhao et al., 2015). Members of the SAR86 clade were positively labeled with ^13^C in all three Syn DOM treatments (Table 4). Members of the SAR86 clade demonstrate enhanced abilities to break down lipids and carbohydrates via copious tonB-dependent receptors (Dupont et al., 2012; Berg et al., 2018), indicating that specialization in targeting distinct DOM quality. SAR86’s carbon assimilation pathways may facilitate its members to exploit DOM resources less accessible to other microbes. *Hyphomonadaceae* often persist in the oligotrophic ocean and are capable of assimilating carbon sources like sugar and organic acids (Abraham et al., 2013; Bayer et al., 2019). Our study showed their potential role in utilizing PPL-altered diatom-derived DOM. Members of the SAR11 subgroup II and SAR202 clades are enriched in the mesopelagic and often bloom shortly after deep convective mixing in the Sargasso Sea (Morris et al., 2005; Carlson et al., 2009; Treusch et al., 2009), suggesting they might utilize components of surface DOM that resists or escapes microbial degradation but is consumed after export from surface to the mesopelagic. Our data showed that SAR11 II and SAR202 clade 1, clade 2, and clade 3 incorporated phytoplankton-derived and PPL-altered DOM. These results are consistent with studies that used single-amplified genomes of SAR202 subgroup 3 to show that they can encode multiple families of oxidative enzymes involved in oxidation of recalcitrant DOM (Landry et al., 2017). *Acidimicrobiales* that were incorporators of TW PPL-DOM in our study previously were shown to be capable of utilizing complex carbon sources (Wang et al., 2019). *Bdellovibrionaceae* are predatory bacteria; their incorporation of ^13^C DOM may be attributed to cross-feeding on prey that have already incorporated DOM (Morris et al., 2002; Orsi et al., 2016), but verifying this will require further investigation. Our results also showed that several rare taxa, including members of *Salinisphaerales*, *Rickettsiales*, and *Anaerolineaceae*, were capable of taking up phytoplankton-derived DOM. Members of these taxonomic groups are found in various marine environments, but their DOM utilization strategies have not been investigated in detail (Hayajawa, 2012; Teske et al., 2013; Angly et al., 2016). This study implies that these rare taxa might also contribute to the microbial processing or transformation of dissolved compounds. It was surprising to find that some taxa that are typically considered to be chemoautotrophs, such as the SAR324 clade, *Nitrospinaceae*, and members of Thaumarchaeota i.e., archaea marine benthic group A, showed ^13^C-DOM incorporation in this study. While we cannot rule out the possibility of “cross feeding,” several studies have reported flexible and opportunistic metabolic lifestyle of these chemoautotrophs: SAR324 contained genes for C1 metabolism (Swan et al., 2011); a genomic study revealed genes for complete oxidation of sulfur-containing organic carbon substrates in *Nitrospinaceae* (Friedrich, 2002); archaea have been shown to be capable of oxidizing urea, polyamine and lignin in microbial remineralization experiments, or taking up ^13^C or ^15^N labeled urea or ^3^H leucine in SIP and FISH studies (Baltar et al., 2010; Connelly et al., 2014; Orsi et al., 2016; Damashek et al., 2019; McDonald et al., 2019; Seyler et al., 2019), although other studies showed that Thaumarchaeota did not assimilate organic carbon (Dekas et al., 2019). Urea concentrations in our Syn exudate_*PPL*_ extract were indeed more than two-fold greater than concentrations measured in the Syn lysate and Syn lysate_*PPL*_ extract and might explain the enriched ^13^C signal resolved in the archaeal biomass only in Syn exudate_*PPL*_ treatment.

Many oligotrophs that incorporated PPL-altered DOM such as members of the SAR11 deep clade, SAR202, SAR86, *Salinisphaeraceae*, and *Acidimicrobiales* clades, were also found to become enriched in the mesopelagic zone during or shortly after annual convective mixing at BATS site in the Sargasso Sea (Figure 10). Our field observations relied on 16S rRNA gene sequence amplification using the V1V2 primers instead of V4 primers as they were part of an extended historical time series data set collected at BATS since the 1990’s that has used this primer set. As such we cannot rule out slight bias between the two primer sets (Parada et al., 2016); however, overall community and ecological dynamics determined from V1V2 and V4 primer sets have been shown to be consistent and robust (Wear et al., 2018). In a direct comparison of a vertical profile collected at BATS amplified with V1V2 and V4 primers (excluding Archaea sequences from V4 primer data as V1V2 primer targets only bacteria) (Klindworth et al., 2013), the relative abundance of SAR11 clade or *Salinisphaeraceae* was underestimated using V4 primer; however, the relative distribution patterns among those taxa across the water column was consistent despite varying primer sets (Supplementary Figure 6). The relative abundance change of specific groups of field time series (V1V2) were compared with experimental data (V4) at the broad family level (Figure 10), and were interpreted as robust comparisons. Oligotrophs dominate the bacterioplankton community in the Sargasso Sea, accounting for over 60−90% of the whole community, whereas copiotrophs are rare members of the free living bacterioplankton community that bloom in response to episodic disturbances (Morris et al., 2005; Treusch et al., 2009; Vergin et al., 2013b). In the Sargasso Sea, phytoplankton blooms in the late winter/early spring result in net DOM accumulation. The DOM that accumulates during the stratified periods from late spring-autumn period resists rapid microbial degradation in the surface (semi-labile, semi-refractory DOM) (Carlson et al., 2002, 2004), and a portion of that seasonally produced DOC is redistributed and mixed into the mesopelagic during winter convective mixing (Carlson et al., 1994; Hansell and Carlson, 2001). The surface-derived DOM that is exported to the mesopelagic zone appears to stimulate a response of mesopelagic oligotrophs i.e., SAR11 II, SAR202 as well as others (Carlson et al., 2009; Treusch et al., 2009). Our SIP study establishes a connection between experimental and environmental observation data, corroborating the important role of some oligotrophs in utilization of transformed/recalcitrant DOM in the Sargasso Sea.

The percentage of ^13^C-labeling varied among different incorporator microbes, leading to differences in mean densities between taxa and treatments (Figure 9). This heterogeneity reflects different microbial activities during incubation. DNA-SIP labeling only occurs when a microbial taxon incorporates the substrate into its DNA at a rate that is sufficiently rapid to increase cell density to above detection limits within the incubation time period. Higher mean density of a microbial taxon indicates that it is likely more active in incorporating a certain substrate, and different metabolic activities among taxa can be attributed to various factors like microbial life history and environmental conditions (Morando and Capone, 2016). For example, for the same TW lysate_*PPL*_ substrate, incorporators differed between the surface and mesopelagic environments, suggesting that DOM uptake might be dependent on initial conditions such as the availability of inorganic macro- or micro- nutrients, DOM quality and quantity, and the resident microbial assemblages that differ between the surface and mesopelagic zones. Clusters of microbial groups across substrates (rows) in the heat map (Figure 9) could be explained not only by different metabolic activities, but also distinct ecological strategies for substrate utilization among bacterioplankton. While generalists can use a variety of organic substrates, specialists target specific or a limited range of substrates (Moran et al., 2003; Alonso and Pernthaler, 2006; Mou et al., 2008). Bacterioplankton incorporating TW DOM and amino acids are consistent with a generalist strategy because they appear to incorporate multiple DOM sources from model compounds to TW-derived DOM. However, bacterioplankton that incorporated Syn-derived DOM appeared to be more specialized, showing exclusivity to this source of DOM (Figure 9 and Table 4). Whether bacteria are generalists or specialists is not often constrained to phylogeny alone. For example, Mayali et al. (2015) demonstrated that operational taxonomic unit (OTU) members of *Oceanospirillales*, *Alteromonadales*, and *Flavobacteriales* were generalists in terms of assimilating various organic matter including protein, lipid and polysaccharide while other OTUs belonging to the same orders were considered specialists and not capable of taking up all tested organic substrates. We observed both generalists and specialists in the *Alphaproteobacteria* or *Gammaproteobacteria* class (Figure 9), indicating intra-clade functional heterogeneity. To get better insight of the different strategies within a bacterioplankton family, a genomic perspective should be considered to confirm the functional potential at the ecotype or species level.

## Conclusion and Implications

The present study identified a wide diversity of copiotrophs and oligotrophs capable of incorporating phytoplankton-derived DOM in the Sargasso Sea. This is the first prototype study demonstrating that solid phase extraction techniques such as that with PPL cartridges can alter DOM quality and therefore influence types of microbial taxa that can respond to and incorporate such DOM. Oligotrophs were dominant incorporators of PPL-altered DOM, in contrast to copiotrophs that dominated the response and incorporation of labile model DOM compounds. These results suggest that DOM extraction on PPL cartridges retained relatively more recalcitrant DOM. Linking DOM varying quality (bioavailability) with distinct bacterioplankton taxa incorporators implies different ecological strategies and niche partitioning among bacterioplankton. The SPE combined with DNA-SIP approach in this study can be readily applied to study bacterioplankton utilization of a diversity of DOM substrates with varying quality and origin in the future.

Our study may help to identify which phytoplankton derived DOM sources and which pretreatments (i.e., PPL extraction) might enrich for targeted oligotrophs in extinction culturing approaches used to isolate bacterioplankton (Connon and Giovannoni, 2002; Giovannoni and Stingl, 2007; Overmann et al., 2017). Cultivation approaches that utilize marine broth media with high concentration organic matter and nutrients (ZoBell, 1941) are often overwhelmed with copiotrophs. Bioavailable organic matter in natural open ocean seawater, where oligotrophs are more prevalent, is at least two orders of magnitude lower than common marine broth media. With the development of extinction cultivation approaches that utilize low-nutrient media, more oligotrophs have been cultivated (Connon and Giovannoni, 2002; Rappe et al., 2002; Giovannoni and Stingl, 2007; Overmann et al., 2017). The SPE-DOM triggered response of a diverse group of oligotrophs reported here indicates that the addition of SPE-DOM in a similar experimental design may be useful to enriching targeted oligotrophs prior to initiating extinction cultivation techniques. Also, the addition of PPL-altered DOM may be a potential organic source in culture media to facilitate selection of oligotrophs in extinction cultivation approaches when culturing time is extended beyond the generation time of these slow-growers.

## Data Availability Statement

The datasets presented in this study can be found in online repositories. The names of the repository/repositories and accession number(s) can be found in the article/ Supplementary Material. Sequence data in this study can be found in NCBI SRA under project PRJNA577154. Other data in this study can be found in https://github.com/shutingliu/SIP_ms_FMICB_2020.git.

## Author Contributions

SL and CC conceived and designed the experiments and wrote the manuscript. SL, NB, JC, KO, CE, and CC conducted the experiments. SL, KO, EH, RP, SG, and LB analyzed the samples. SL, KO, LB, CN, KV, and CC did the data analysis. All authors reviewed and contributed to the manuscript writing.

## Conflict of Interest

KV was employed by the Microbial DNA Analytics. The remaining authors declare that the research was conducted in the absence of any commercial or financial relationships that could be construed as a potential conflict of interest.
