# Supplementary material for: Stable Isotope Probing Identifies Bacterioplankton Lineages Capable of Utilizing Dissolved Organic Matter Across a Range of Bioavailability
Source: Front Microbiol. 2020 Oct 7;11:580397. doi: 10.3389/fmicb.2020.580397 (PMC7575717; doi:10.3389/fmicb.2020.580397)
Supplement: Supplementary file 1 [file Data_Sheet_1.pdf]

(A)

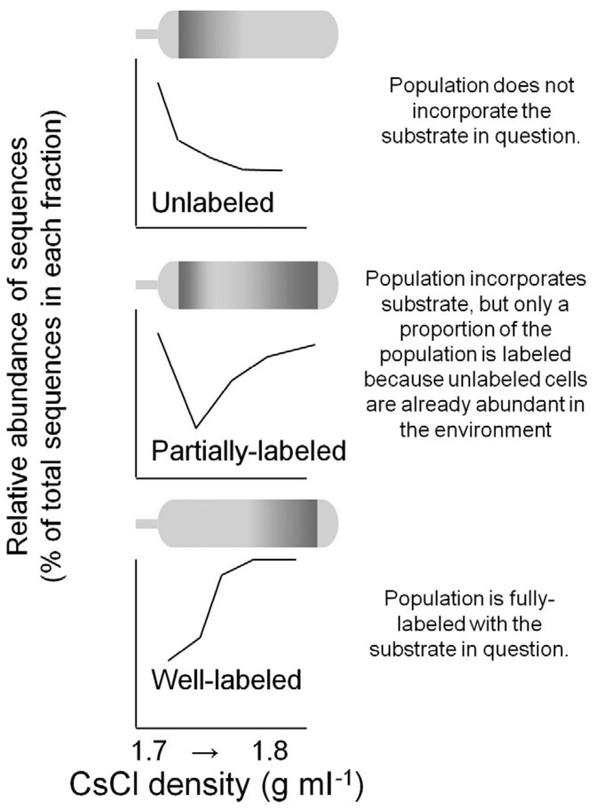
Relative abundance of sequences (% of total sequences in each fraction)
Unlabeled
Population does not incorporate the substrate in question.
Partially-labeled
Population incorporates substrate, but only a proportion of the population is labeled because unlabeled cells are already abundant in the environment
Well-labeled
Population is fully-labeled with the substrate in question.
1.7 → 1.8
CsCl density (g ml-1)

(B)

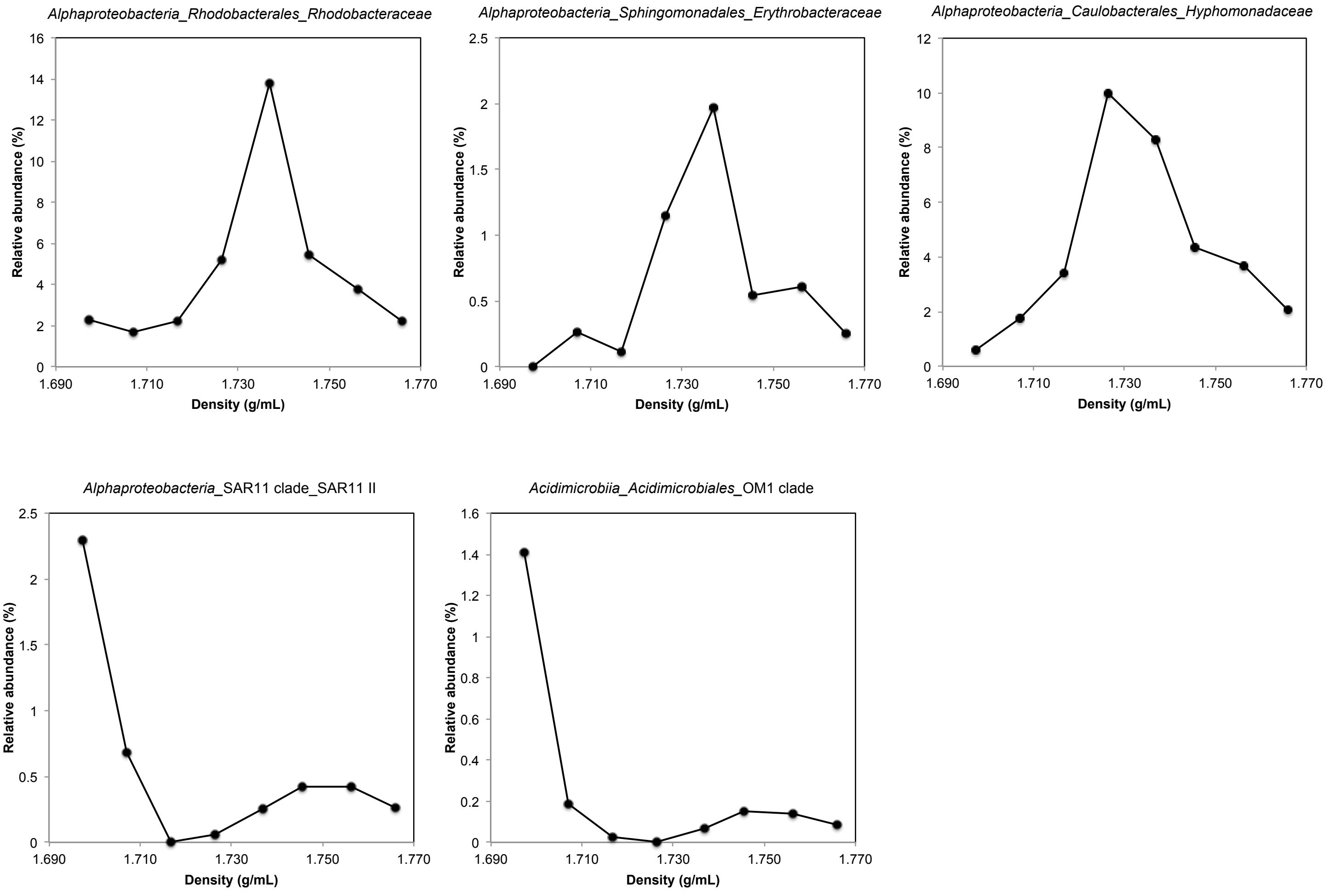
Alphaproteobacteria_Rhodobacterales_Rhodobacteraceae
Alphaproteobacteria_Sphingomonadales_Erythrobacteraceae
Alphaproteobacteria_Caulobacterales_Hyphomonadaceae
Alphaproteobacteria_SAR11 clade_SAR11 II
Acidimicrobiia_Acidimicrobiales_OM1 clade
Relative abundance (%)
Density (g/mL)
1.690
1.710
1.730
1.750
1.770

(C)

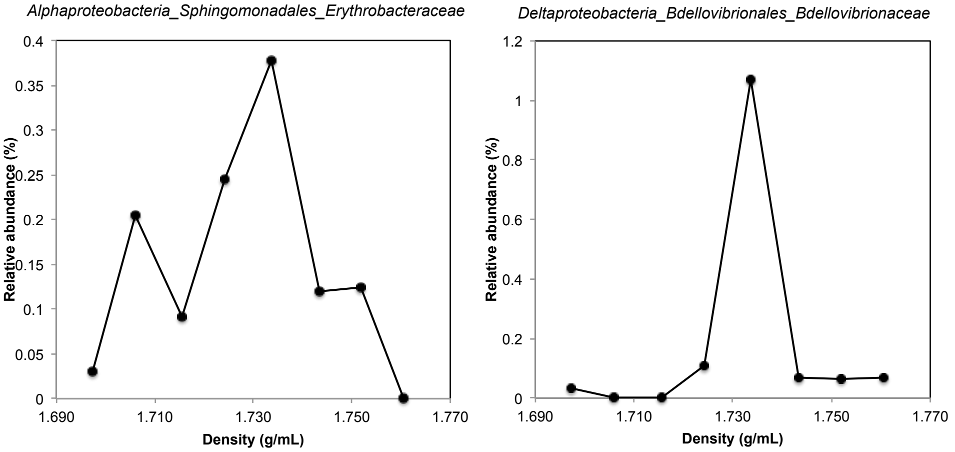
Alphaproteobacteria_Sphingomonadales_Erythrobacteraceae
Relative abundance (%)
Density (g/mL)
Deltaproteobacteria_Bdellovibrionales_Bdellovibrionaceae
Relative abundance (%)
Density (g/mL)

(D)

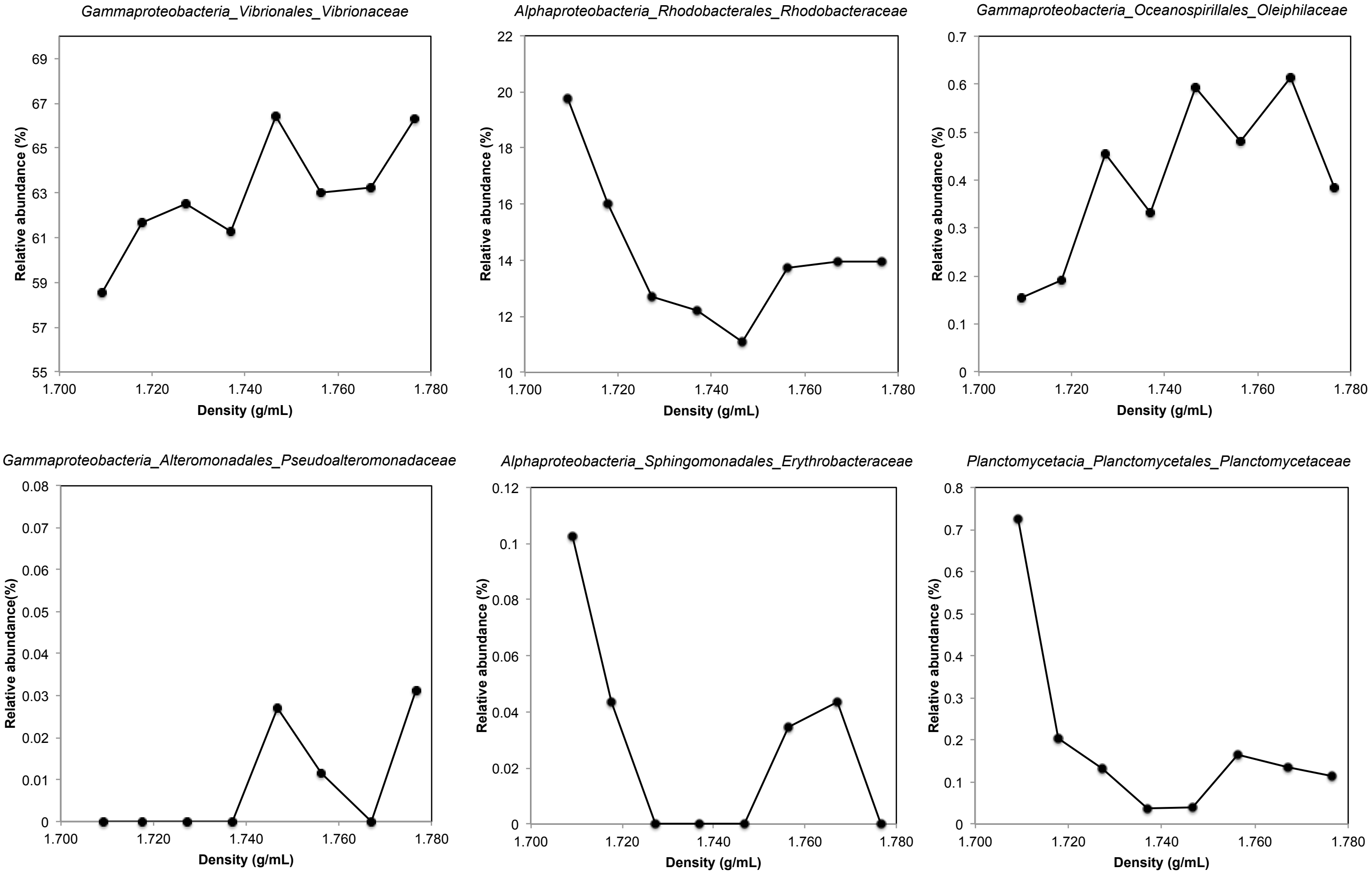
Gammaproteobacteria_Vibrionales_Vibrionaceae
Relative abundance (%)
Density (g/mL)
Alphaproteobacteria_Rhodobacterales_Rhodobacteraceae
Relative abundance (%)
Density (g/mL)
Gammaproteobacteria_Oceanospirillales_Oleiphilaceae
Relative abundance (%)
Density (g/mL)
Gammaproteobacteria_Alteromonadales_Pseudoalteromonadaceae
Relative abundance(%)
Density (g/mL)
Alphaproteobacteria_Sphingomonadales_Erythrobacteraceae
Relative abundance (%)
Density (g/mL)
Planctomycetacia_Planctomycetales_Planctomycetaceae
Relative abundance (%)
Density (g/mL)

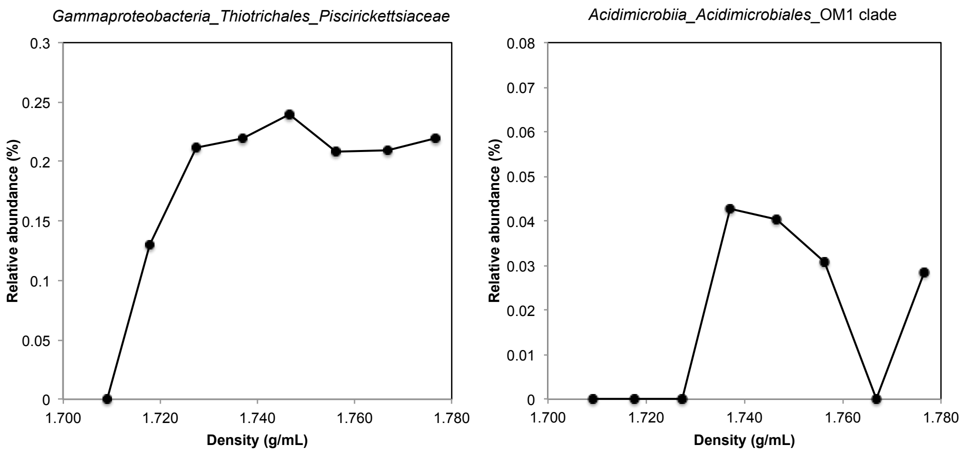
Gammaproteobacteria_Thiotrichales_Piscirickettsiaceae
Acidimicrobiia_Acidimicrobiales_OM1 clade
Relative abundance (%)
Density (g/mL)

(E)

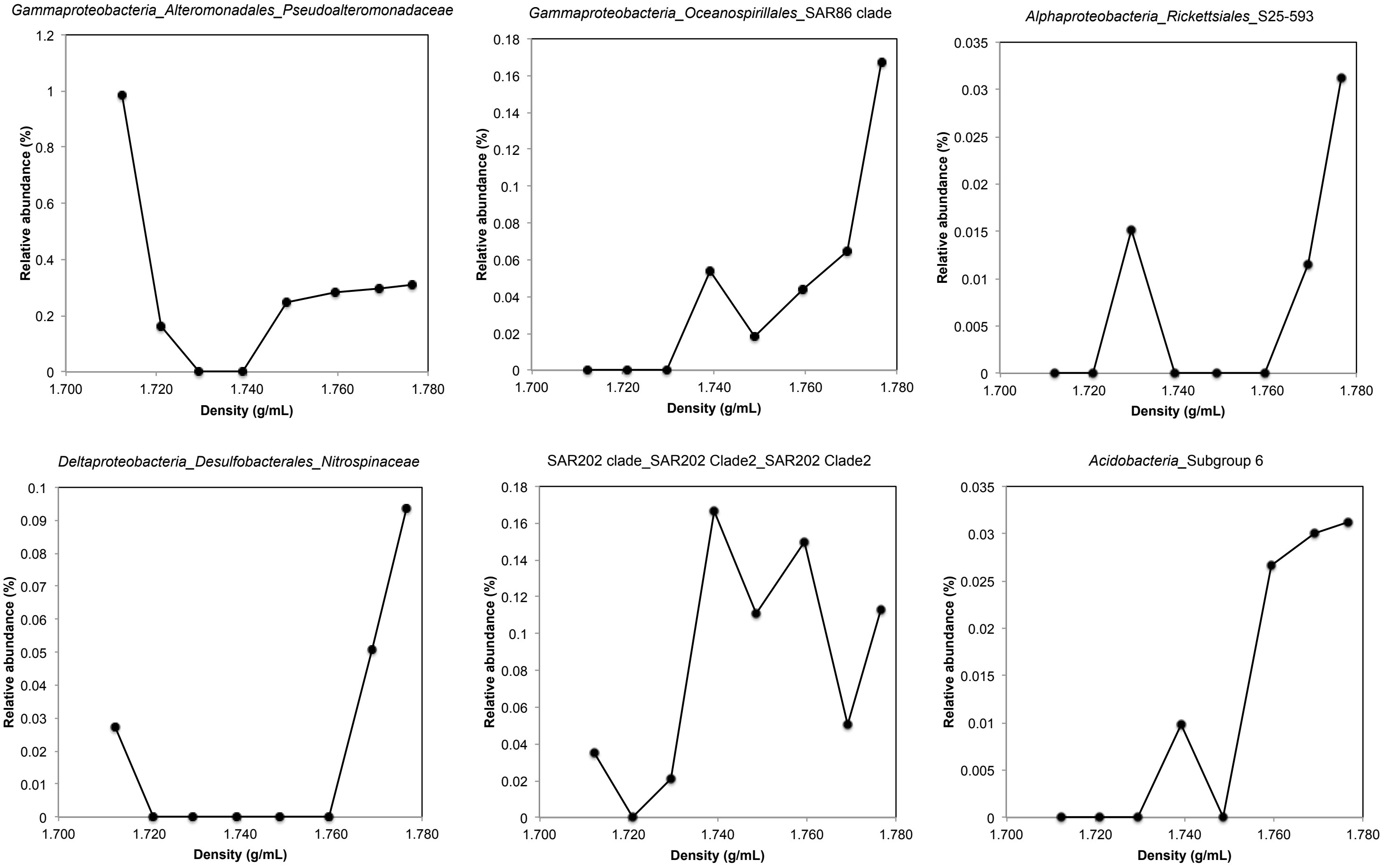
Gammaproteobacteria_Alteromonadales_Pseudoalteromonadaceae
Gammaproteobacteria_Oceanospirillales_SAR86 clade
Alphaproteobacteria_Rickettsiales_S25-593
Deltaproteobacteria_Desulfobacterales_Nitrospinaceae
SAR202 clade_SAR202 Clade2_SAR202 Clade2
Acidobacteria_Subgroup 6
Relative abundance (%)
Density (g/mL)

(F)

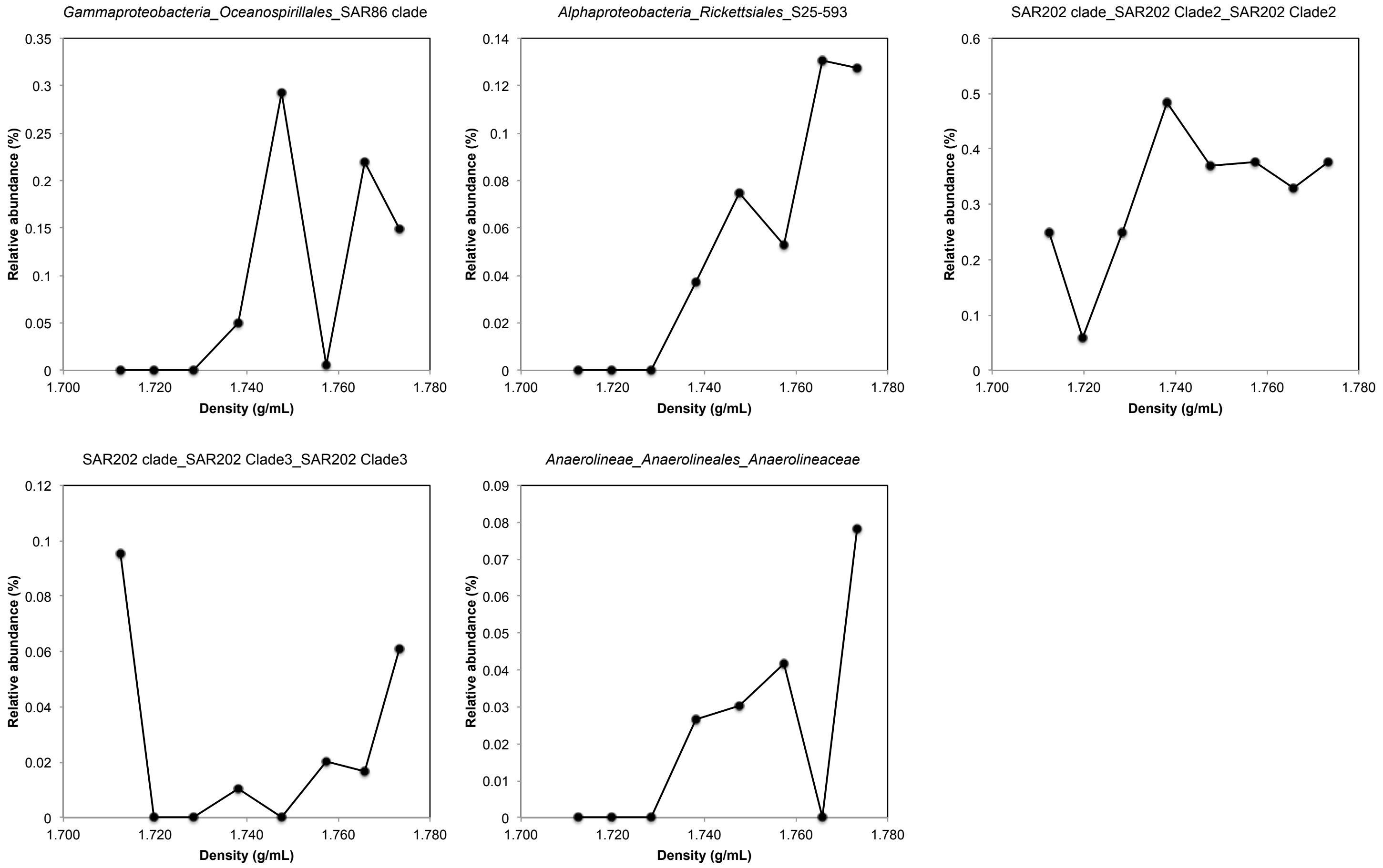

*Gammaproteobacteria_Oceanospirillales_*SAR86 clade
*Alphaproteobacteria_Rickettsiales_*S25-593
SAR202 clade_SAR202 Clade2_SAR202 Clade2
SAR202 clade_SAR202 Clade3_SAR202 Clade3
Anaerolineae_Anaerolineales_Anaerolineaceae
Relative abundance (%)
Density (g/mL)
1.700
1.720
1.740
1.760
1.780

(G)

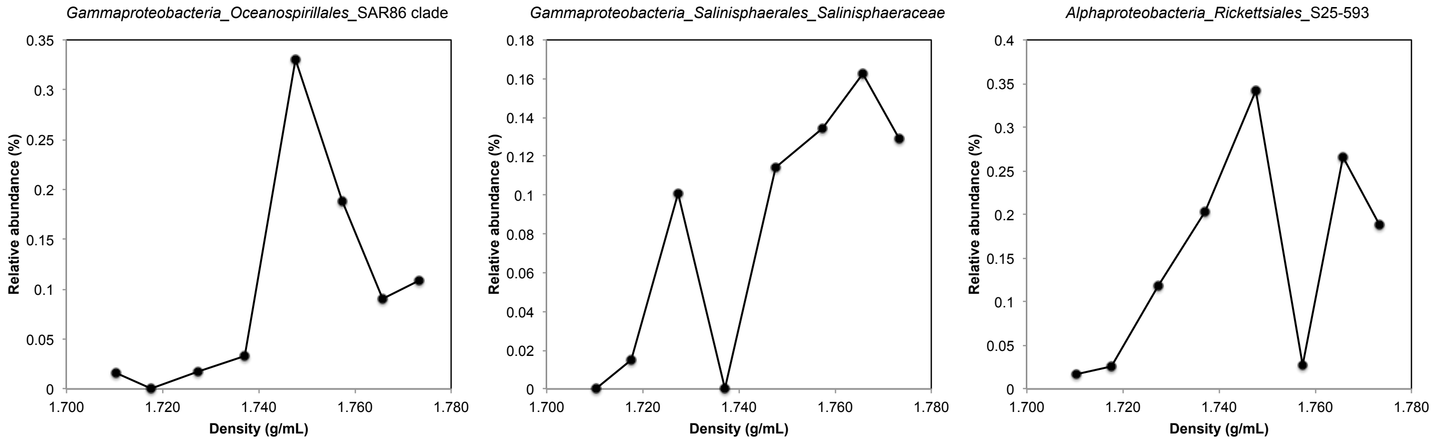

*Gammaproteobacteria_Oceanospirillales_*SAR86 clade
Gammaproteobacteria_Salinisphaerales_Salinisphaeraceae
*Alphaproteobacteria_Rickettsiales_*S25-593
Relative abundance (%)
Density (g/mL)
1.700
1.720
1.740
1.760
1.780

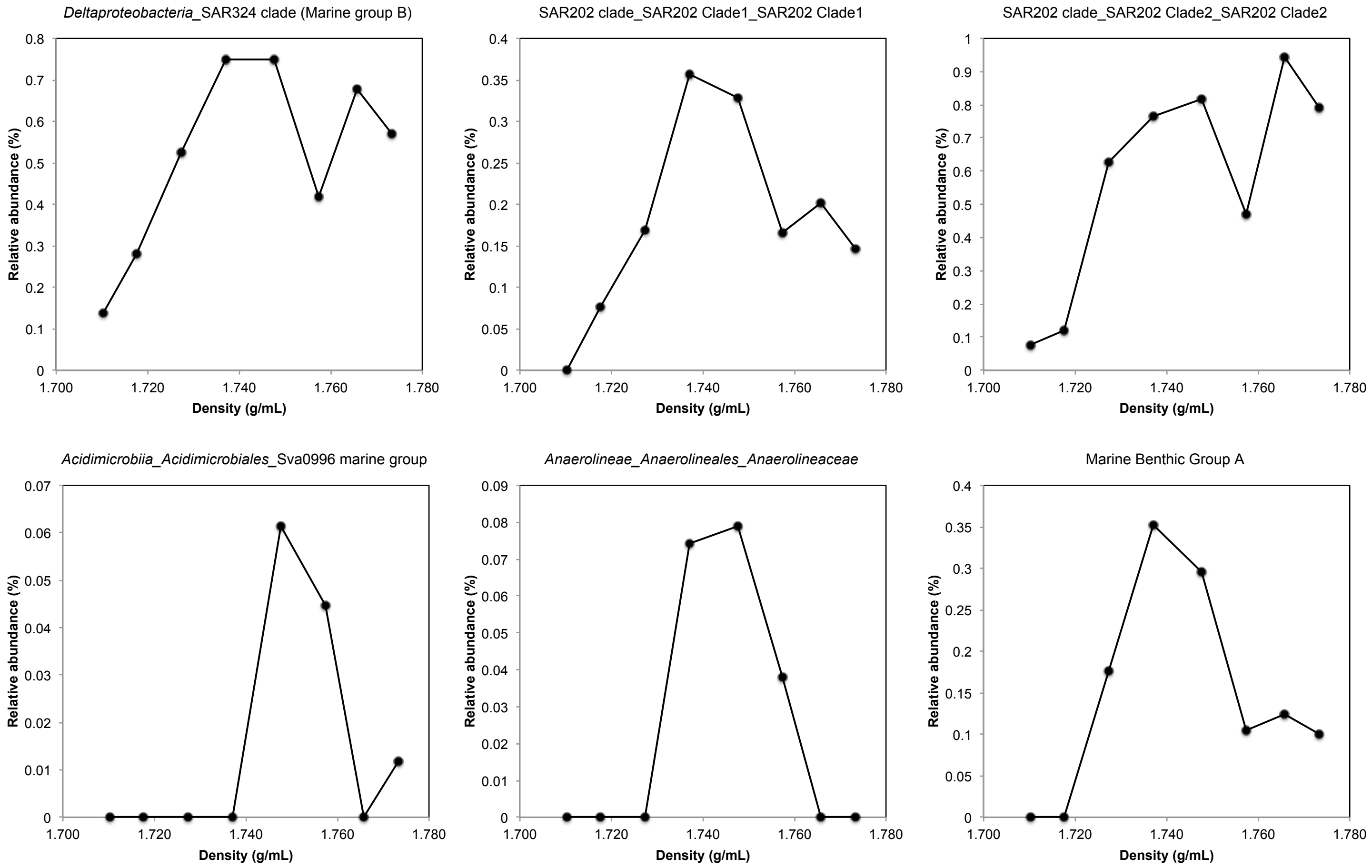

Figure S1. (A) Theoretical examples of relative abundance across density fractions for unlabeled, partially-labeled, and ^{13}C well-labeled taxa (Figure adapted from Nelson and Carlson, 2012). (B-G) Empirical examples of relative abundance across density fractions for partially-labeled and ^{13}C well-labeled taxa in the (B) S TW lysate $_{PPL}$, (C) M TW lysate $_{PPL}$, (D) M Amino acid, (E) M Syn lysate, (F) M Syn lysate $_{PPL}$, and (G) M Syn exudate $_{PPL}$ treatments.

(A)

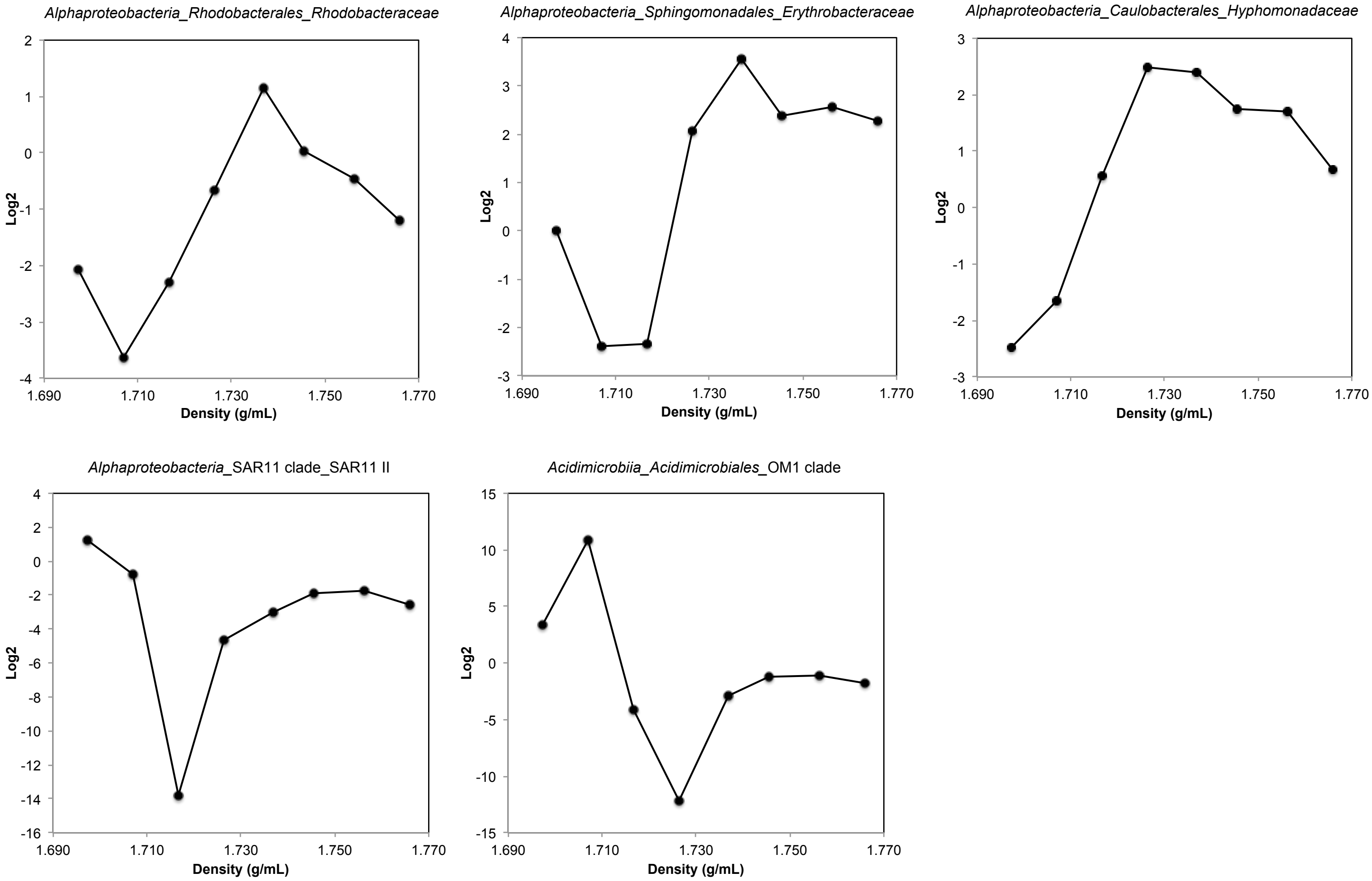

Alphaproteobacteria_Rhodobacterales_Rhodobacteraceae
Alphaproteobacteria_Sphingomonadales_Erythrobacteraceae
Alphaproteobacteria_Caulobacterales_Hyphomonadaceae
*Alphaproteobacteria*_SAR11 clade_SAR11 II
*Acidimicrobiia_Acidimicrobiales*_OM1 clade
Log2
Density (g/mL)
1.690 1.710 1.730 1.750 1.770

(B)

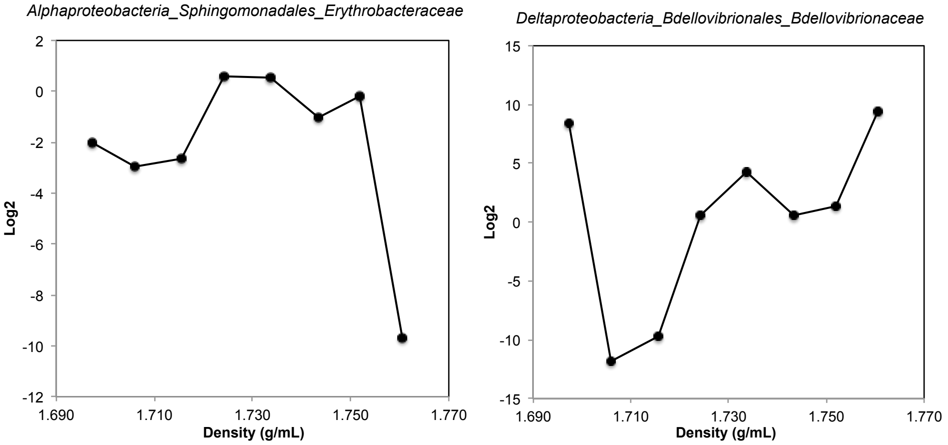

Alphaproteobacteria_Sphingomonadales_Erythrobacteraceae
Deltaproteobacteria_Bdellovibrionales_Bdellovibrionaceae
Log2
Density (g/mL)
1.690 1.710 1.730 1.750 1.770

(C)

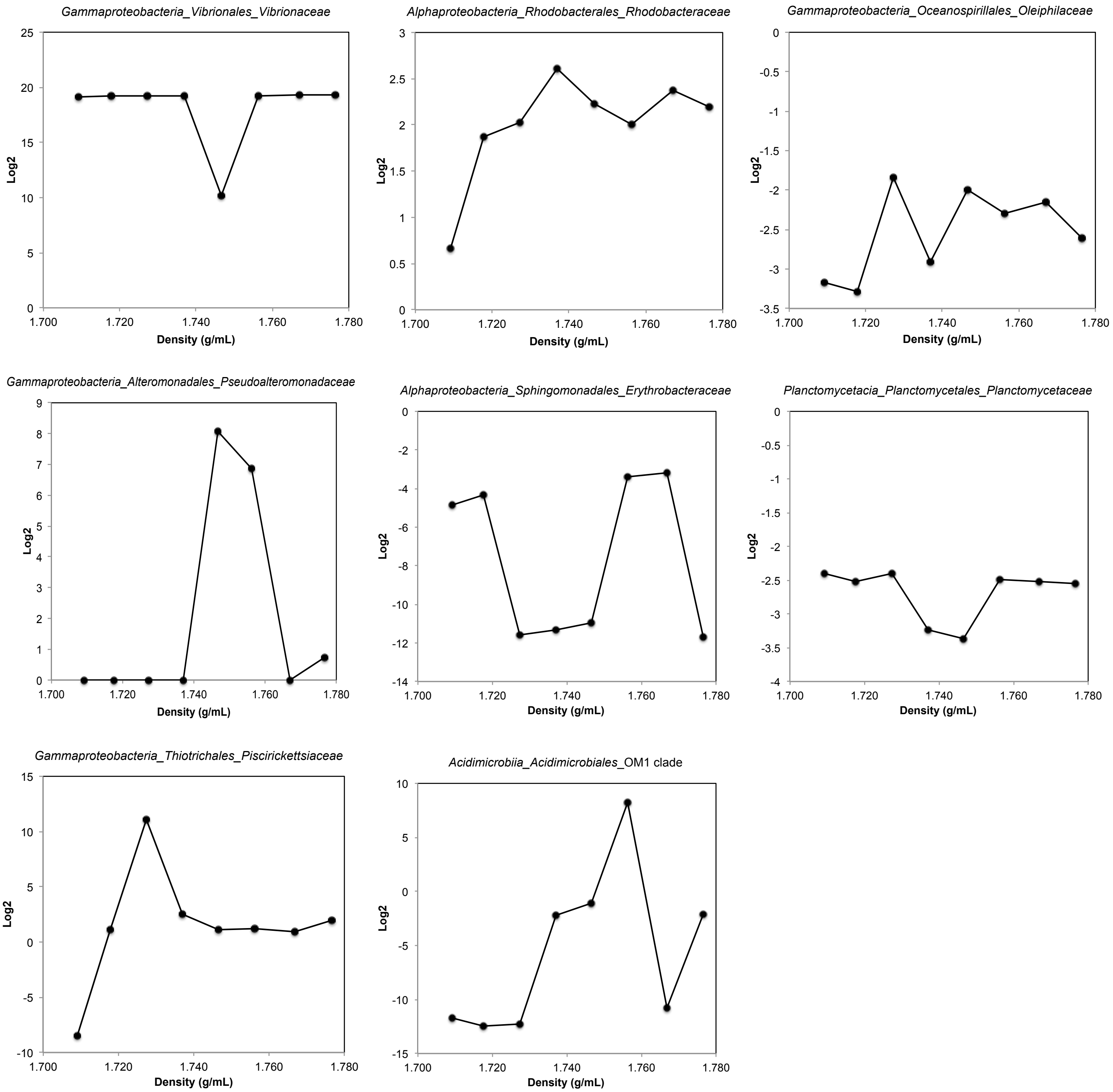
Gammaproteobacteria_Vibrionales_Vibrionaceae
25
20
15
10
5
0
Log2
1.700
1.720
1.740
1.760
1.780
Density (g/mL)
Alphaproteobacteria_Rhodobacterales_Rhodobacteraceae
3
2.5
2
1.5
1
0.5
0
Log2
1.700
1.720
1.740
1.760
1.780
Density (g/mL)
Gammaproteobacteria_Oceanospirillales_Oleiphilaceae
0
-0.5
-1
-1.5
-2
-2.5
-3
-3.5
Log2
1.700
1.720
1.740
1.760
1.780
Density (g/mL)
Gammaproteobacteria_Alteromonadales_Pseudoalteromonadaceae
9
8
7
6
5
4
3
2
1
0
Log2
1.700
1.720
1.740
1.760
1.780
Density (g/mL)
Alphaproteobacteria_Sphingomonadales_Erythrobacteraceae
0
-2
-4
-6
-8
-10
-12
-14
Log2
1.700
1.720
1.740
1.760
1.780
Density (g/mL)
Planctomycetacia_Planctomycetales_Planctomycetaceae
0
-0.5
-1
-1.5
-2
-2.5
-3
-3.5
-4
Log2
1.700
1.720
1.740
1.760
1.780
Density (g/mL)
Gammaproteobacteria_Thiotrichales_Piscirickettsiaceae
15
10
5
0
-5
-10
Log2
1.700
1.720
1.740
1.760
1.780
Density (g/mL)
Acidimicrobiia_Acidimicrobiales_OM1 clade
10
5
0
-5
-10
-15
Log2
1.700
1.720
1.740
1.760
1.780
Density (g/mL)

(D)

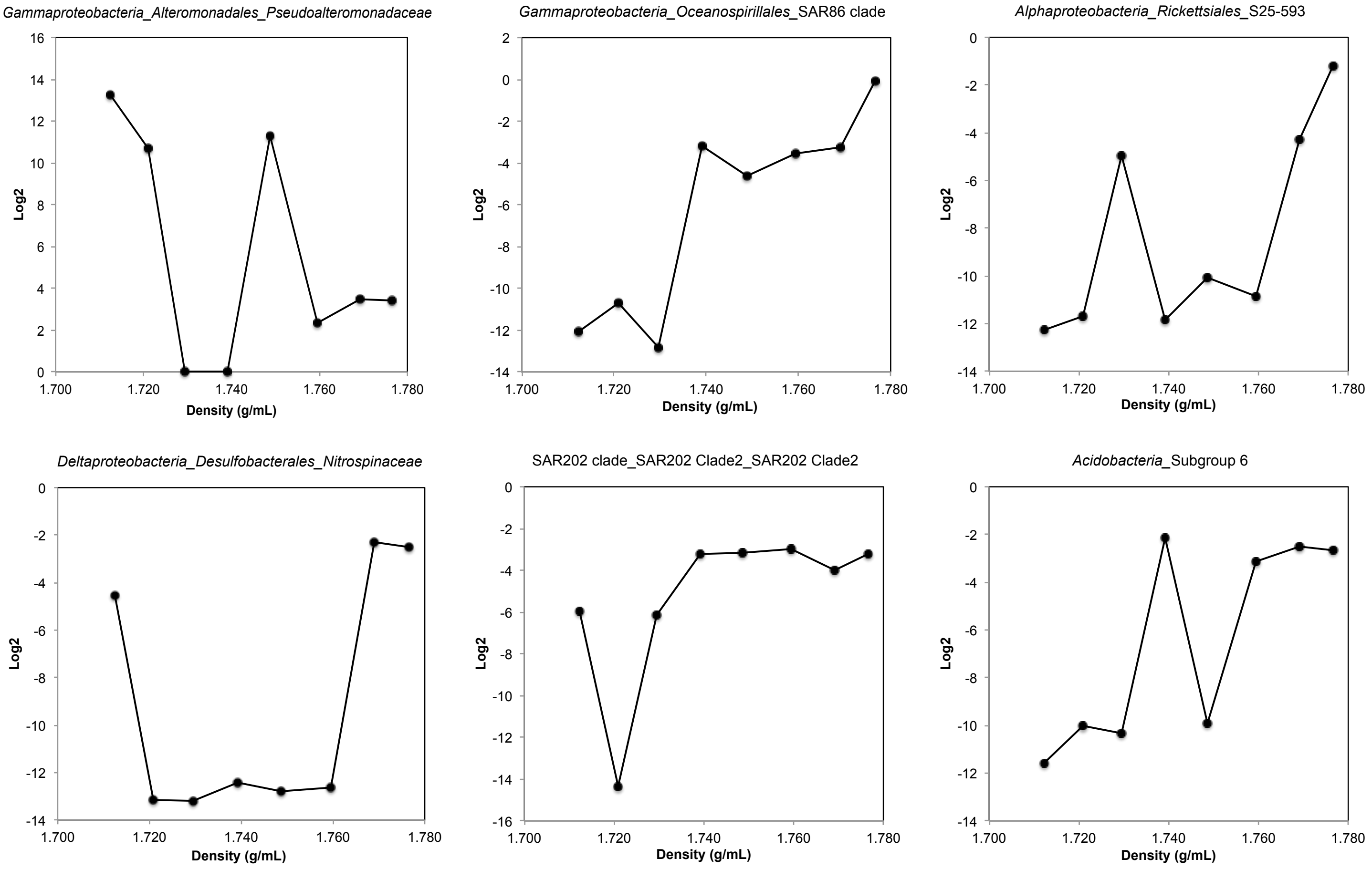

Gammaproteobacteria_Alteromonadales_Pseudoalteromonadaceae
Gammaproteobacteria_Oceanospirillales_SAR86 clade
Alphaproteobacteria_Rickettsiales_S25-593
Deltaproteobacteria_Desulfobacterales_Nitrospinaceae
SAR202 clade_SAR202 Clade2_SAR202 Clade2
Acidobacteria_Subgroup 6
Log2
Density (g/mL)

(E)

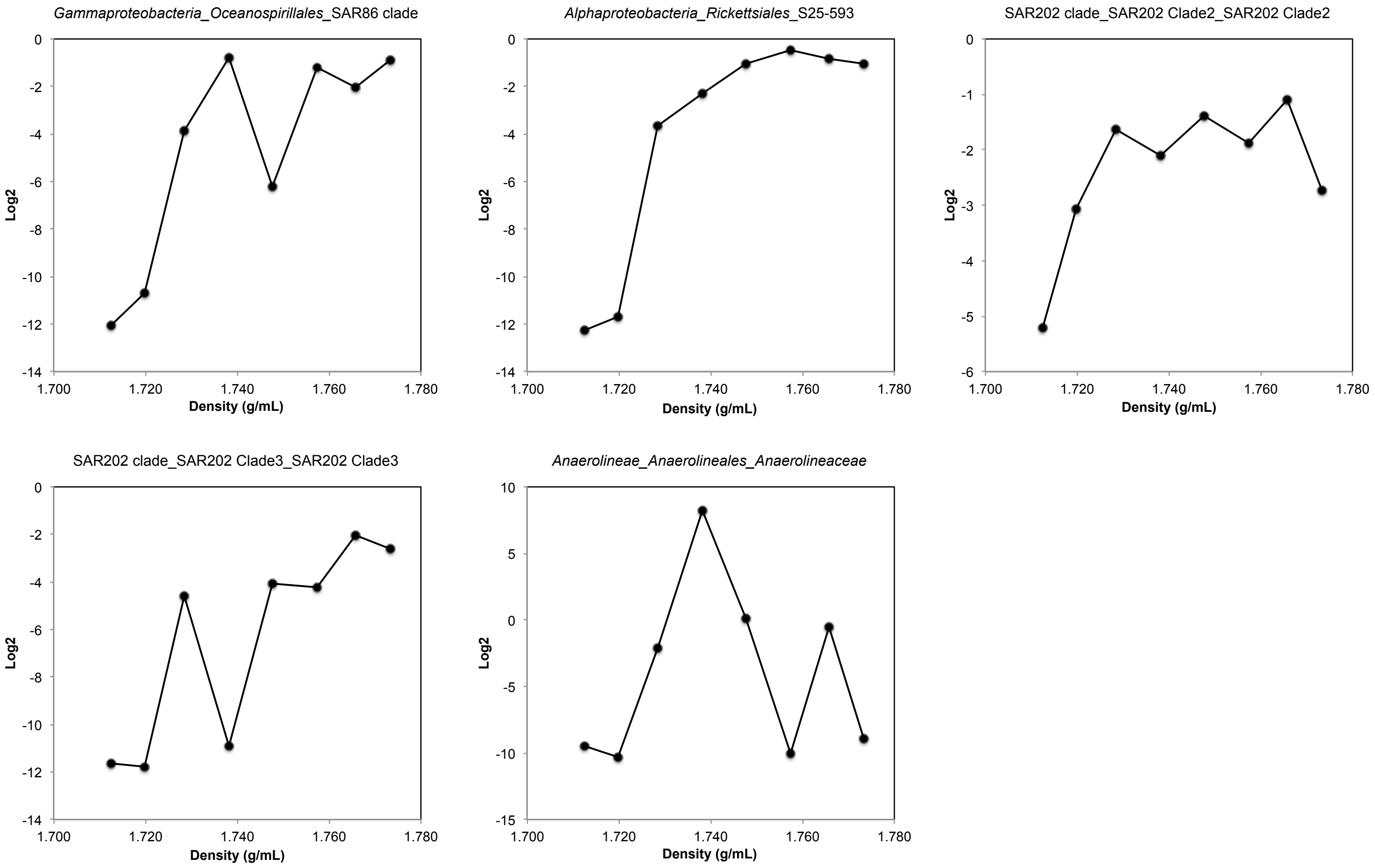

Gammaproteobacteria_Oceanospirillales_SAR86 clade
Alphaproteobacteria_Rickettsiales_S25-593
SAR202 clade_SAR202 Clade2_SAR202 Clade2
SAR202 clade_SAR202 Clade3_SAR202 Clade3
Anaerolineae_Anaerolineales_Anaerolineaceae
Log2
Density (g/mL)

(F)

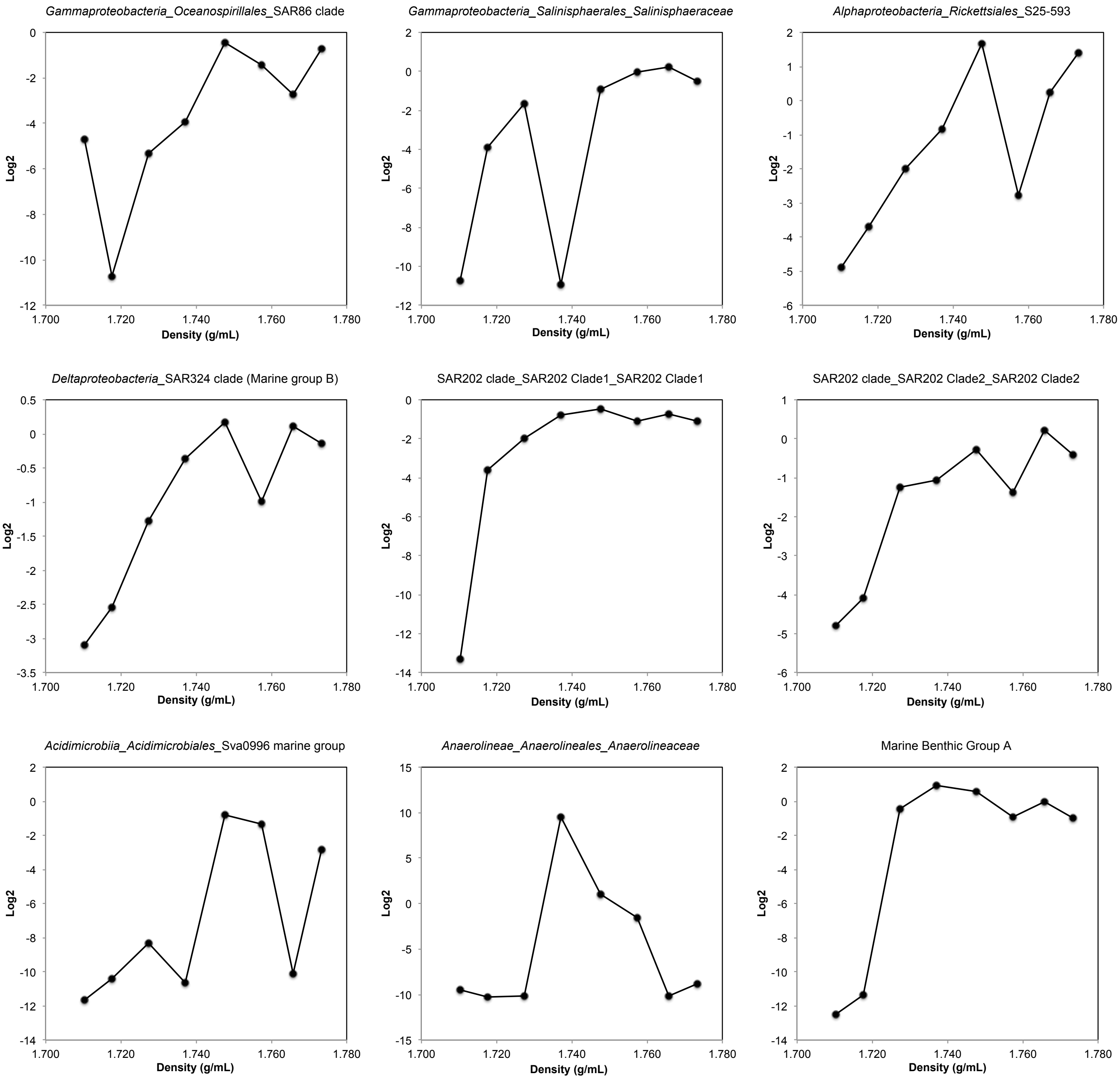

Figure S2. Log2 ratios of relative abundance between amendments and unamended controls across density fractions for partially-labeled and ^{13}C well-labeled taxa in the (A) S TW lysate $_{PPL}$, (B) M TW lysate $_{PPL}$, (C) M Amino acid, (D) M Syn lysate, (E) M Syn lysate $_{PPL}$, and (F) M Syn exudate $_{PPL}$ treatments.

(A)

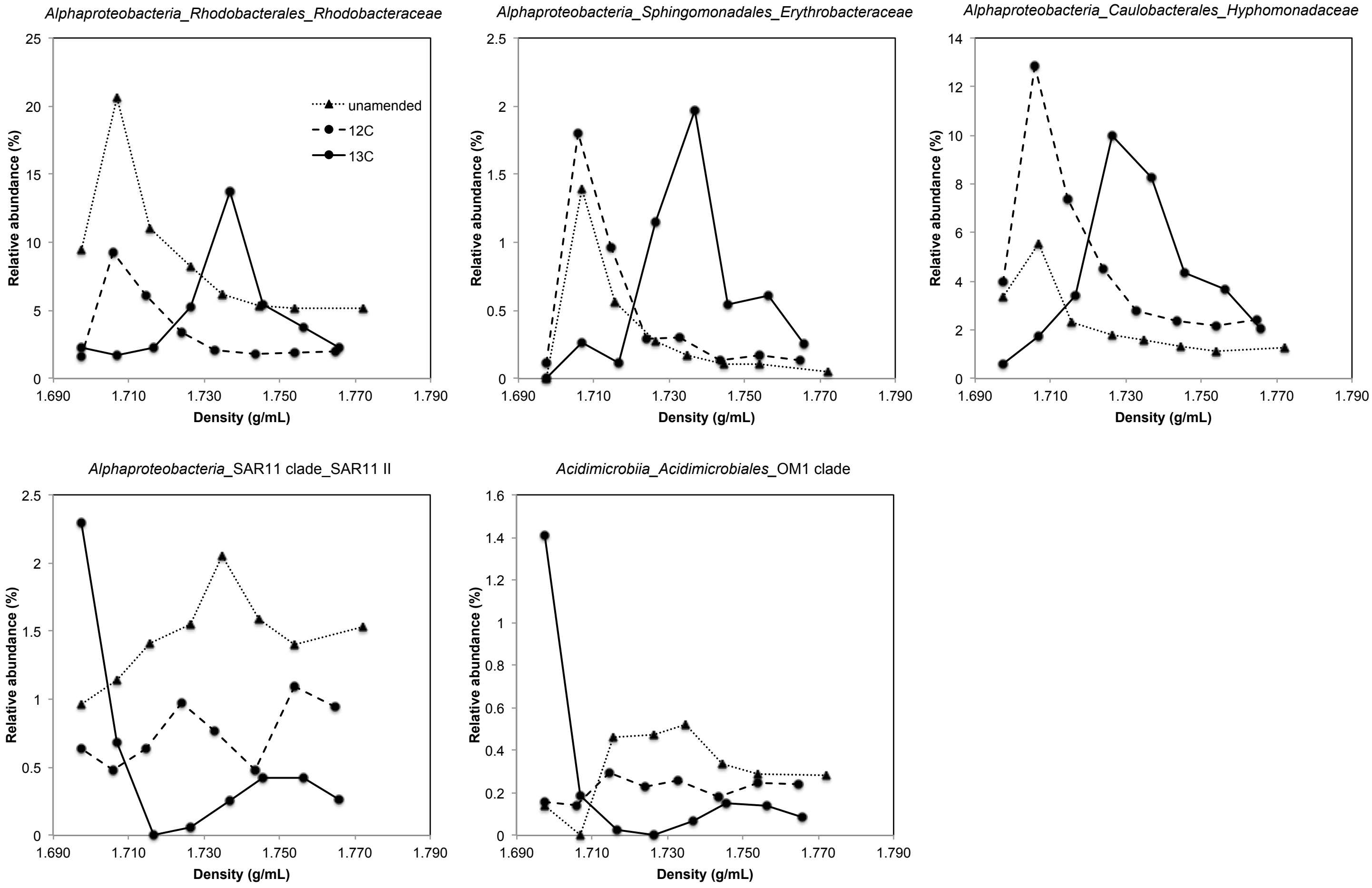

(B)

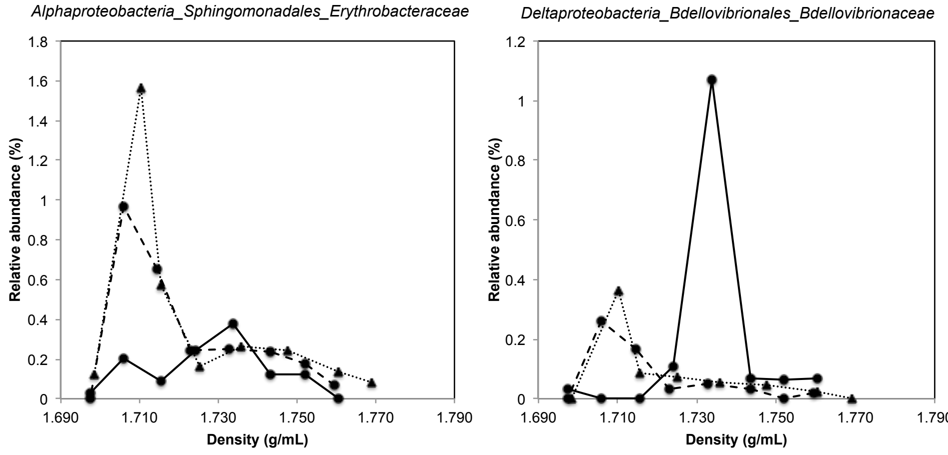

Figure S3. Relative abundance across density fractions for partially-labeled and ^{13}C well-labeled taxa in the unamended control, ^{12}C TW lysate $_{PPL}$ vs. ^{13}C TW lysate $_{PPL}$ treatments in the July 2016 (A) surface and (B) mesopelagic incubations.

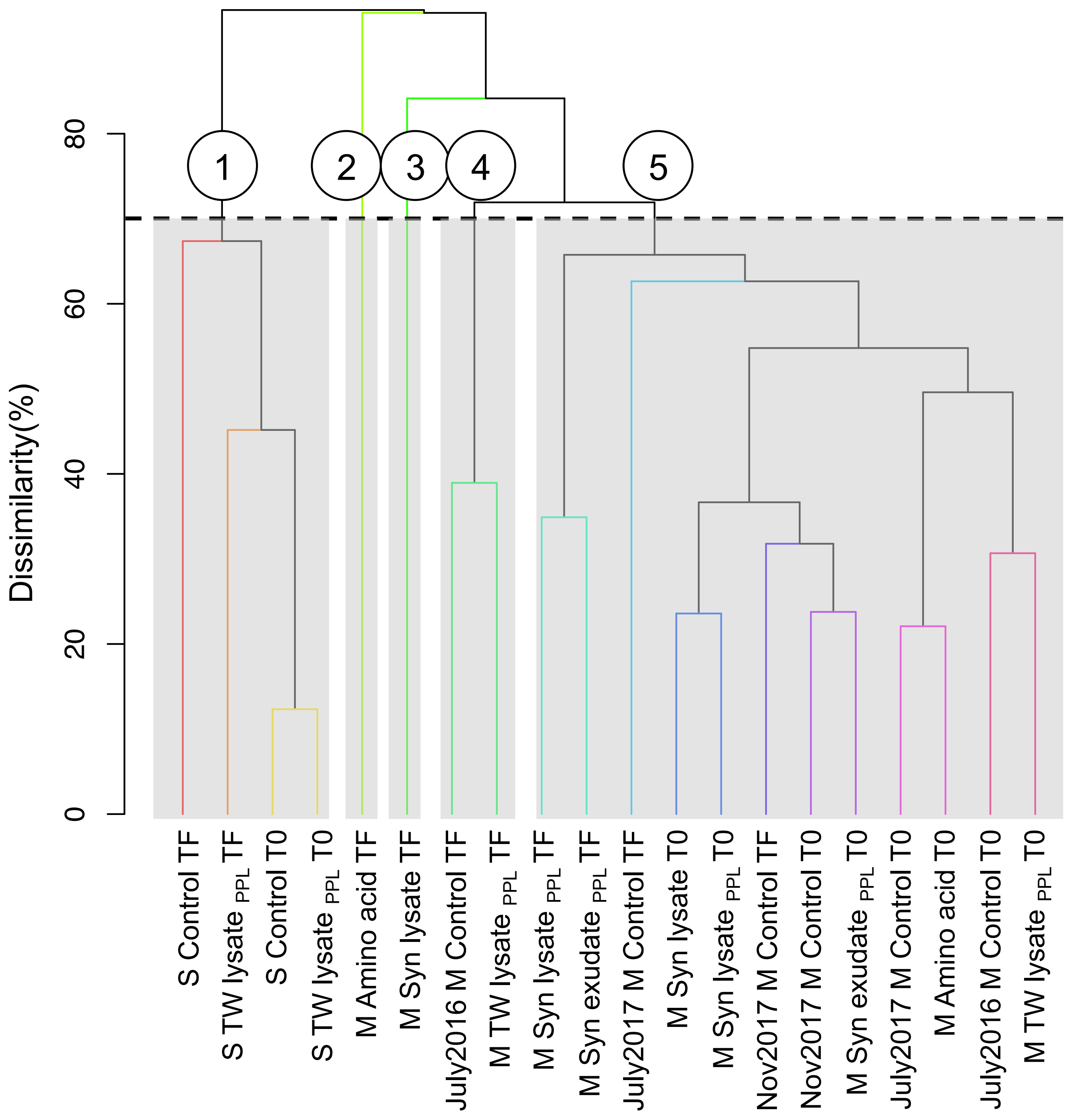

Figure S4. Hierarchical clustering using simprof analysis (α=0.05) on unfractionated samples based on Bray-Curtis distance (*Alteromonadaceae* excluded to avoid bias by its dominance at TF samples). Five clusters (grey box and numbers in circle) were identified based on a threshold of 70% dissimilarity.

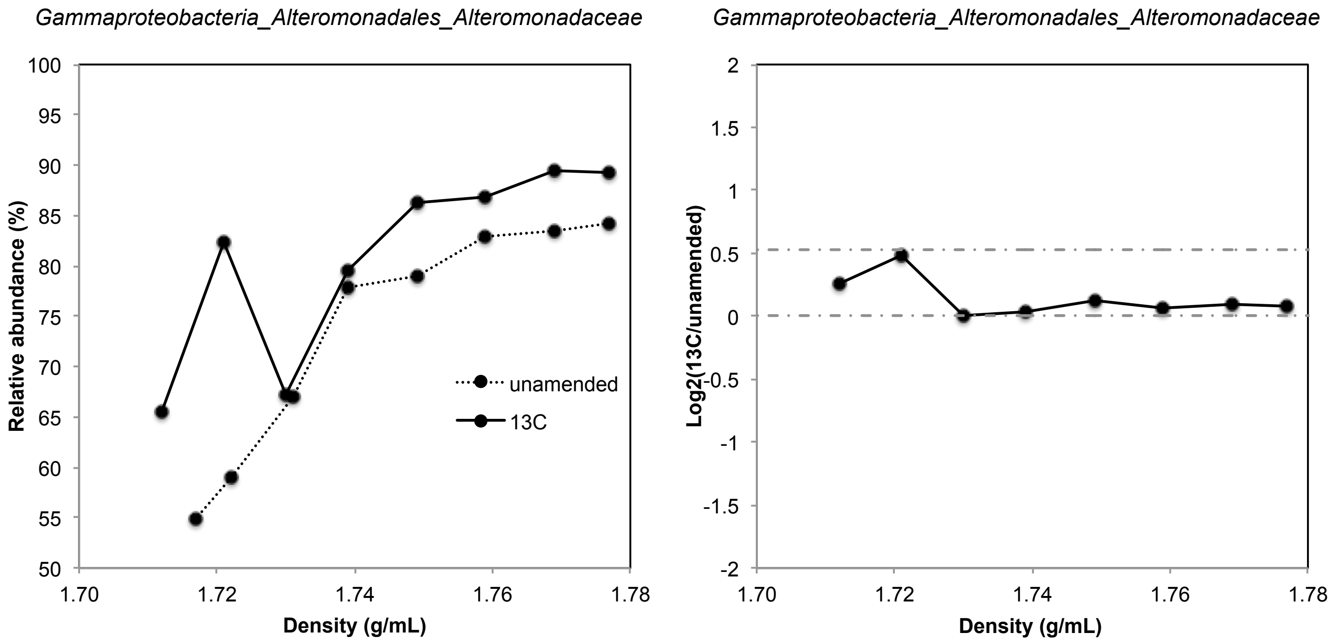

Figure S5. An example of false positive taxa that showed labeling in relative abundance vs. density plot but are actually unlabeled after normalizing to unamended control. Threshold of 0.53 (±log2(1+20%), as 20% of taxa needed to be labeled for confident identification) between lowest and highest log2 value was shown in dashed lines in log2 plot. Fluctuations within threshold lines were considered as non-significant and taxa were identified as unlabeled.

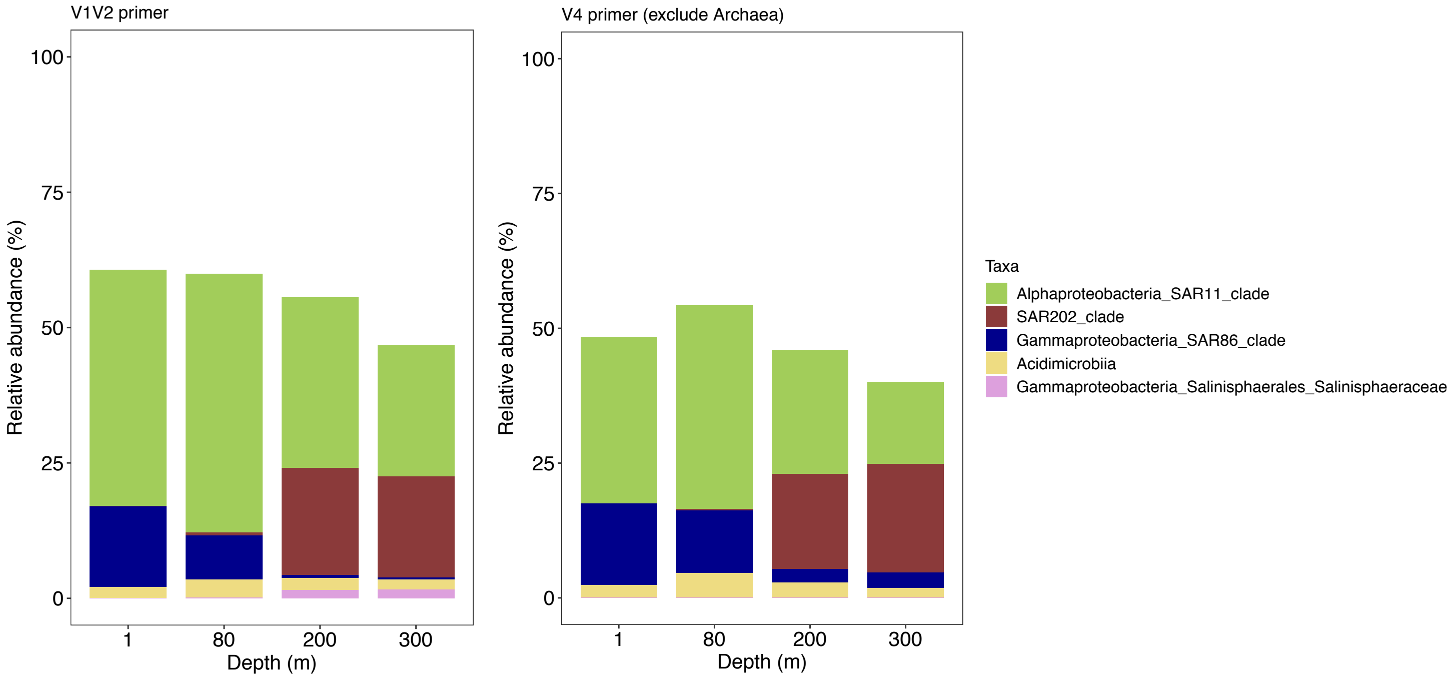

Figure S6. Comparison of relative abundance for selected taxa from a 16S rRNA gene dataset in June 2015 at BATS using V1V2 primer vs. V4 primer (Archaea sequences were excluded before calculating relative abundance in V4 primer dataset).
